# Tetra-*O*-Methyl Nordihydroguaiaretic Acid Broadly Suppresses Cancer Metabolism and Synergistically Induces Strong Anticancer Activity in Combination with Etoposide, Rapamycin and UCN-01

**DOI:** 10.1371/journal.pone.0148685

**Published:** 2016-02-17

**Authors:** Kotohiko Kimura, Ru Chih C. Huang

**Affiliations:** Department of Biology, Johns Hopkins University, Baltimore, Maryland, United States of America; Academia Sinica, TAIWAN

## Abstract

The ability of Tetra-*O*-methyl nordihydroguaiaretic acid (M_4_N) to induce rapid cell death in combination with Etoposide, Rapamycin, or UCN-01 was examined in LNCaP cells, both in cell culture and animal experiments. Mice treated with M_4_N drug combinations with either Etoposide or Rapamycin showed no evidence of tumor and had a 100% survival rate 100 days after tumor implantation. By comparison all other vehicles or single drug treated mice failed to survive longer than 30 days after implantation. This synergistic improvement of anticancer effect was also confirmed in more than 20 cancer cell lines. In LNCaP cells, M_4_N was found to reduce cellular ATP content, and suppress NDUFS1 expression while inducing hyperpolarization of mitochondrial membrane potential. M_4_N-treated cells lacked autophagy with reduced expression of BNIP3 and ATG5. To understand the mechanisms of this anticancer activity of M_4_N, the effect of this drug on three cancer cell lines (LNCaP, AsPC-1, and L428 cells) was further examined via transcriptome and metabolomics analyses. Metabolomic results showed that there were reductions of 26 metabolites essential for energy generation and/or production of cellular components in common with these three cell lines following 8 hours of M_4_N treatment. Deep RNA sequencing analysis demonstrated that there were sixteen genes whose expressions were found to be modulated following 6 hours of M_4_N treatment similarly in these three cell lines. Six out of these 16 genes were functionally related to the 26 metabolites described above. One of these up-regulated genes encodes for CHAC1, a key enzyme affecting the stress pathways through its degradation of glutathione. In fact M_4_N was found to suppress glutathione content and induce reactive oxygen species production. The data overall indicate that M_4_N has profound specific negative impacts on a wide range of cancer metabolisms supporting the use of M_4_N combination for cancer treatments.

## Introduction

**Fig 1:**
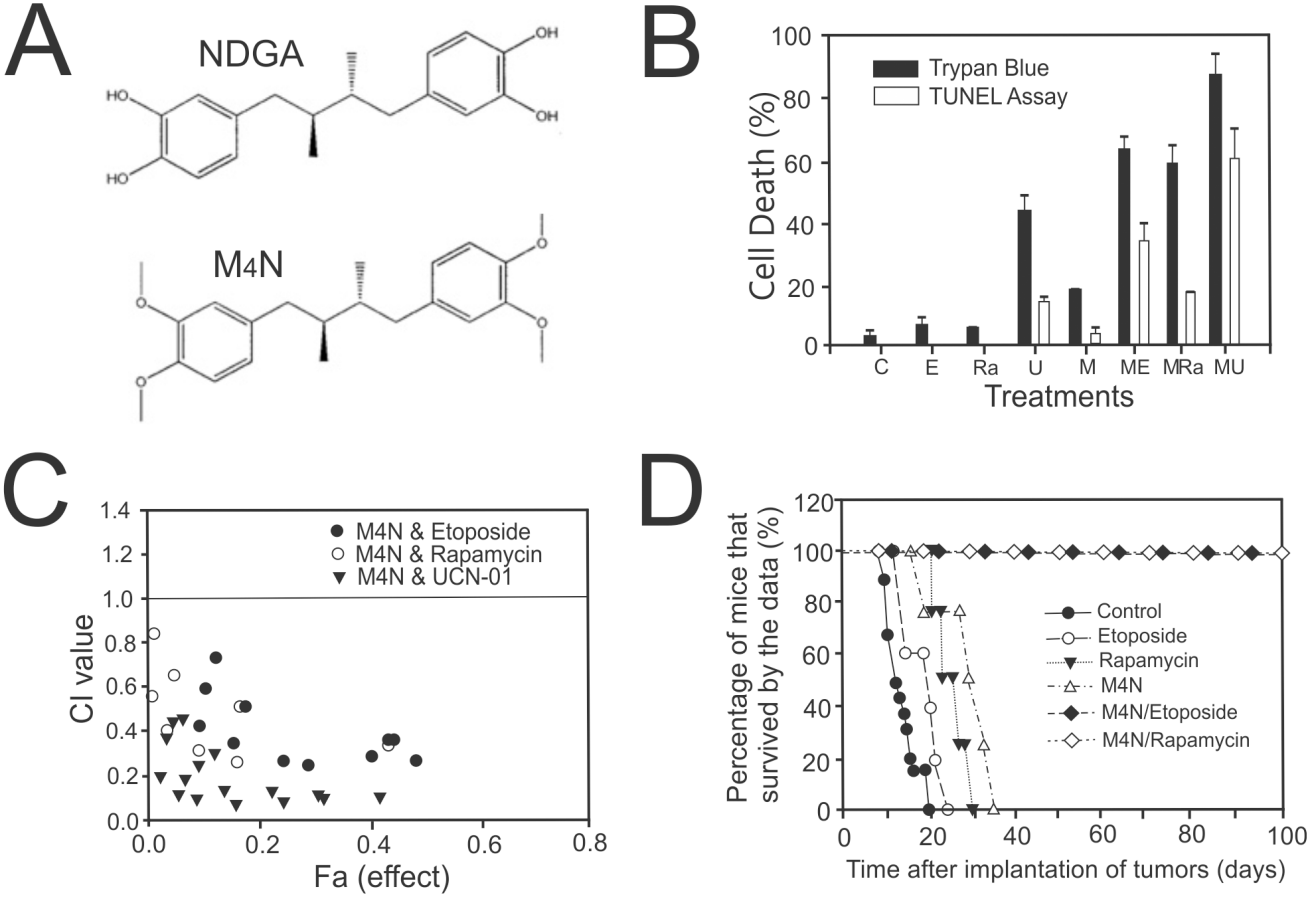
Synergistic induction of anticancer activity by M_4_N combination treatments in LNCaP human prostate cancer. **A.** Molecular structures of nordihydroguaiaretic acid (NDGA) and tetra-O-methyl nordihydroguaiaretic acid (M_4_N). **B:** Synergistic cell death induction in LNCaP tissue culture cells. C: control, E: Etoposide (20 μM), Ra: Rapamycin (20 μM), U: UCN-01 (2 μM), M: M_4_N (80 μM), ME: M_4_N+Etoposide, MR: M_4_N+Rapamycin, MU: M_4_N+UCN-01. The cell death was measured by TUNEL assay and Trypan blue exclusion assay at 24 h after treatment. Data are presented as mean±SD in triplicate. **C:** A Chou-Talalay plot for TUNEL-positive cell death induced by combination treatments in LNCaP cells. Combination index (CI) <1, +1, and >1 indicate synergism, additive effect, and antagonism. **D:** Effect of combination treatments of M_4_N with Etoposide or Rapamycin on the survival of nude (nu/nu) mice orthotropically implanted with LNCaP tumors. The percentage of mice that have survived by the date after tumor inoculation was shown for each group.

We have previously reported that tetra-*O*-methyl nordihydroguaiaretic acid (M_4_N), also known as EM1421 and terameprocol, possessed antiviral and anti-cancer activities [1–4] and that M_4_N could be potentially useful as an anticancer drug. We also showed that one of principal pharmacological activities of M_4_N was to inhibit the activity of SP1 transcription factor by binding to GC-rich regions (SP1 consensus sequences) of gene promoters competitively with SP1[4]. It was also found that M_4_N, because of this activity, was able to suppress SP1-regulated *CDK1*expression and to cause cell cycle arrest at the G2 phase of the cell cycle [4], which partially provided an explanation for M_4_N induced anticancer activity, as unregulated growth is considered to be one of hallmarks of cancer. In addition Wang et al. showed that the overexpression of SP1 facilitated development and progression of gastric cancer [5], further suggesting that suppression of SP1-regulated transcription could induce anticancer activities. Other than these studies on transcription inhibition, there have been but a few studies related to the pharmacological activities of M_4_N. Most notably Pardini et al. showed that nordihydroguaiaretic acid (NDGA, a precursor compound of M_4_N) inhibited the mitochondrial electron transport system and suppressed oxidative phosphorylation [6–7]. NDGA was a candidate anticancer agent, however it was eventually proven to be clinically inappropriate due to its significant side effect. Because of the similarity in molecular structure between M_4_N and NDGA (Fig 1A), these data regarding NDGA are relevant to our studies on M_4_N. Overall, these previous findings of M_4_N seem to indicate that M_4_N might have multiple pharmacological activities.

M_4_N is currently in Phase I/II clinical trials in patients with various advanced cancer [8, 9]. The results of these clinical trials so far indicate that M_4_N has a certain degree of anticancer activity, but was unable to induce remission in any of these patients with advanced cancers. One of the most frequently attempted strategy to increase anticancer efficacy of chemotherapy drugs is combination treatment with one or more appropriately selected drugs [10]. An important finding from the clinical trials with M_4_N is that the toxicity of the drug was very low [8, 9]. Patients were able to tolerate high doses of M_4_N with minimal side-effects, which make this drug very suitable for being use in multidrug treatments (for instance the LD_50_ of M_4_N for mice is greater than 1000mg/kg while that of NDGA is only 75mg/kg [11, 12]). The optimization of multidrug treatments requires deep knowledge about pharmacological mechanisms of anticancer actions of the drugs to be used in combination. However, the precise mechanism of anticancer activity of M_4_N is still largely unknown. The difficulty in understanding the pharmacology of M_4_N is rooted in the fact that this drug can be expected to influence multiple biochemical activities since its pharmacological target, SP1 transcription factor, controls a vast number of house-keeping genes [13]. As a matter of fact, a survey of 22,633 known genes in the Ensemble Human Genome database (http://www.ensembl.org/index.html) indicated that just over half (52.5%) contained the SP1 binding motif in their immediate (500bp) upstream regions.

In this study, we first exercised our best efforts to find certain drug combinations with M_4_N which achieved promising anticancer efficacy in tissue culture and mouse xenograft experiments. Through these efforts, we successfully discovered that M_4_N combination treatments with Etoposide and Rapamycin were able to completely eradicate orthotopically implanted LNCaP derived tumors and metastasis in nude mice. The M_4_N combination treated mice had a 100% survival rate 100 days after tumor implantation. By comparison all other vehicles or single drug treated mice failed to survive longer than 30 days after implantation. This illustrated the extraordinary potential ability of M_4_N to improve anticancer efficacy by combination treatments. To understand the pharmacological mechanisms involved, we examined the biochemical and physiological impacts of M_4_N treatment on cancer cells with the assistance of high-throughput screening methods such as GC/LC-MS (gas chromatography/liquid chromatography-mass spectroscopy)-based metabolite assay and deep RNA sequencing. Since, the target molecules of M_4_N could be numerous due to the nature of its pharmacological activity as an SP1 inhibitor, the high-throughput screening with its power to collect information about the status of a great number of metabolites and mRNA at once was very useful to decipher the complex nature of M_4_N activity. Our study so far has revealed that there was a substantial reduction of metabolites essential for tumor growth in all three cancer cell lines studied following a short period of M_4_N treatment, and that synergistic induction of caspase cleavage and rapid reactive oxygen species production occurred when M_4_N was used with second anticancer drugs in combination.

## Results

### Synergistic induction of anticancer effect by M_4_N-based combination treatments

We evaluated the use of M_4_N in multiple drug combination treatments using cell culture experiments and nude mouse xenograft studies. In these studies, we used the LNCaP human prostate cancer cell line as a principal experimental material since it has been used in animal models of metastatic prostate cancer [14]. In addition it has been shown that cancer genetic mutations frequently occurred in the LNCaP cell line [15], suggesting this cell line behaves similarly to cancers in clinical settings which often undergo various gene mutations during their progression [16]. As a pilot experiment to determine the most appropriate drugs to be used in combination with M_4_N for clinical applications, we selected three anticancer drugs, namely Etoposide [17], Rapamycin [18], and UCN-01 [19] and evaluated anticancer efficacy of combination treatments with these drugs in LNCaP cells using both the TUNEL assay and Trypan blue exclusion assay. The results (Fig 1B) showed that M_4_N synergistically induced cell death with all three anticancer drugs examined. A Chou-Talalay plot [20] confirmed that all the combination treatments were greatly synergistic (Fig 1B). In addition, the amount of cell death detected by Trypan blue exclusion assay far exceeded that detected by TUNEL assay (Fig 1A), indicating that TUNEL-negative cell death played a major role in the synergistic cell death, especially in M_4_N combination treatment with Rapamycin. The combination treatments were effective in a panel of cancer cell lines which were chosen for their genetic diversity (S1 Fig). Synergistic death, however, was not observed in HL-1, a normal mouse cardiac muscle cell line (our unpublished data). Dose reduction index (DRI) for LNCaP cells was shown in S2 Fig. The benefit of the combination treatments was also evaluated using xenograft mice bearing LNCaP tumors. The survival rate curve (Fig 1D) indicated that all of the LNCaP tumor–bearing mice that received a combination of M_4_N with Etoposide or Rapamycin survived beyond 100 days after tumor implantation, whereas none of those treated with a single drug survived beyond 34 days due to multiple metastases [21]. Additionally several other xenograft mice experiments confirmed that M_4_N-based combination treatments were effective in many cancer cell lines other than LNCaP cells [22]. The data overall showed a superiority of combination treatments compared to single drug treatments and also it showed that M_4_N seemed to work well with various drugs whose pharmacological mechanisms of their anticancer activity were very different from each other.

### The combined treatment with M_4_N induces caspase-7 cleavage

**Fig 2:**
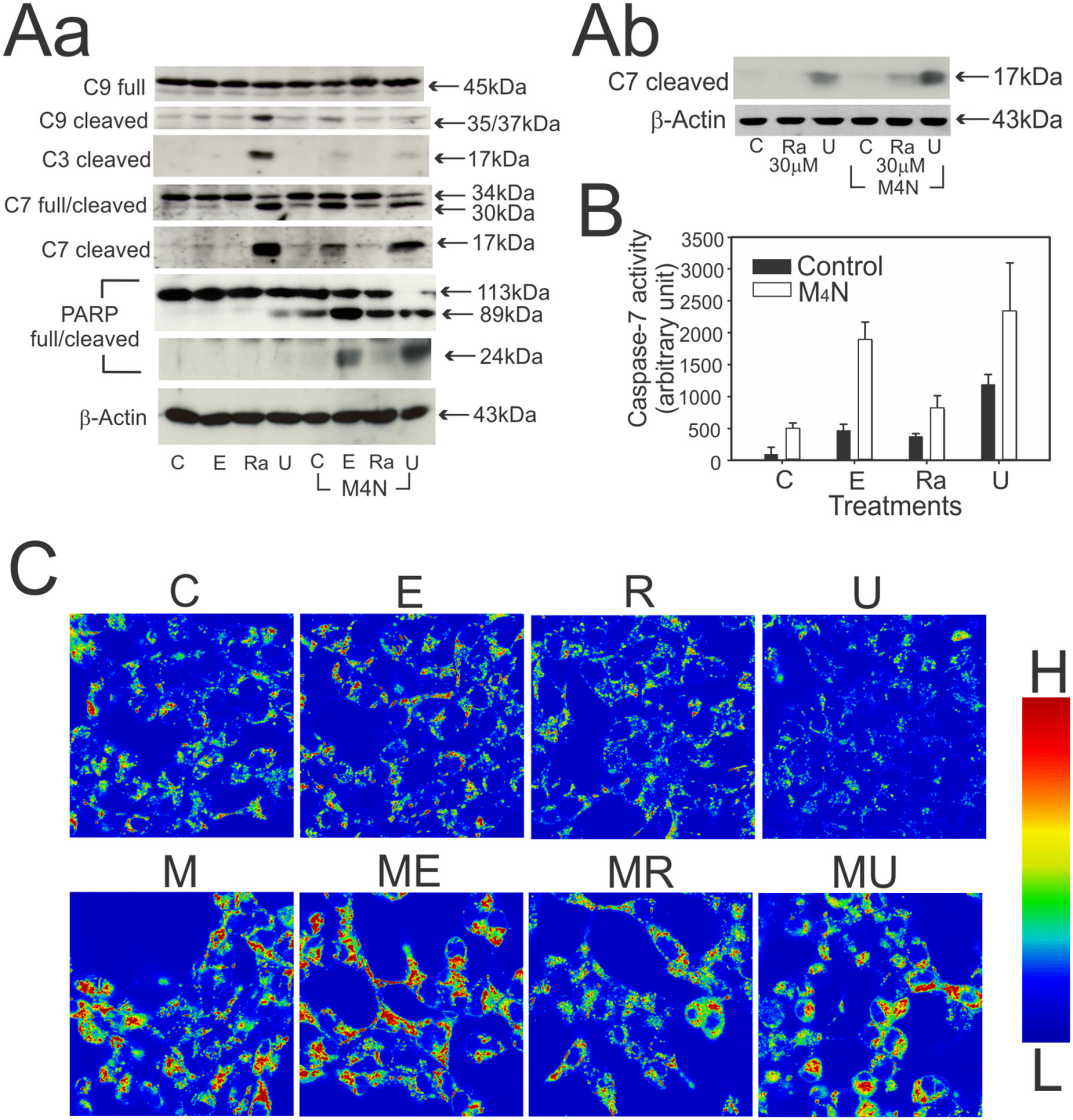
Effect of combination treatments on cell death-related cellular functions. **A-B:** Analysis of caspase-dependent cell death mechanisms induced by combination treatments of M_4_N with Etoposide, Rapamycin, or UCN-01. **Aa & Ab:** Cleavage of caspase-3, -4, -7, and -9 along with poly-ADP ribose polymerase (PARP) in LNCaP cells treated by the combination treatments for 17 h. β-Actin was used as a control. **B:** Caspase-7 enzymatic activity in LNCaP cells treated by the combination treatments for 13 h. Data are presented as mean±SD in triplicate. **A & B:** C: control, E: Etoposide (10 μM), Ra: Rapamycin (10 μM), Ra30μM: Rapamycin (30 μM), U: UCN-01 (2 μM), M4N: M_4_N (80 μM), and Casp: Caspase. **C:** Effect of combination treatments on mitochondrial membrane potential (ΔΨ_m_). LNCaP cells were treated with M_4_N (80 μM) in combination with Etoposide (10 μM), Rapamycin (10 μM), or UCN-01 (2 μM) for 4 h. The ΔΨ_m_ was measured using JC-1 dye. The image shows the ratios obtained by dividing the intensity at 568 nm-excitation light (J-aggregates) by that at 488 nm-excitation light (J-aggregates + monomer). The color bar is shown on the right side of the figure (H: high, L: low ratio).

To explore possible involvement of caspases in cell death mechanisms of M_4_N combination treatments [23], we examined the caspase cleavage profile in LNCaP cells following single-drug (M_4_N, Etoposide, Rapamycin, or UCN-01) and M_4_N combination (M_4_N/Etoposide, M_4_N/Rapamycin, and M_4_N/UCN-01) treatments by Western blot analysis. After single-drug treatments, UCN-01 but not Etoposide or Rapamycin strongly activated caspases-9, -3, and -7 (Fig 2Aa and 2Ab). On the other hand, only a small amount of caspase-9 and -3 activity was detected following any of the M_4_N combination treatments (Fig 2Aa). Apparently, M_4_N interfered with the ability of UCN-01 to activate caspase-9 and -3. On the contrary, the western blot analysis of caspase-7 (Fig 2Aa) demonstrated that M_4_N did not prevent cleavage of this caspase by UCN-01. Moreover, cleavage of caspase-7 also occurred following M_4_N/Etoposide treatment and to a lesser extent following M_4_N/Rapamycin treatment (Fig 2Aa and 2Ab). The colorimetric assay for caspase-7 activity (Fig 2B) confirmed the results of the western blot analysis (Fig 2Aa and 2Ab). These data overall indicated that combination treatments activated caspase-7 much more than any single drug treatment. Interestingly either UCN-01 treatment or any of the M_4_N combination treatments yielded two different caspase-7 fragments, a large (p30) and a small (p17) fragment (Fig 2Aa and 2Ab). Boucher *et al*. showed that one of the main functions of caspase-7 was to cleave and inactivate poly-ADP ribose polymerase (PARP) [24]. For this reason we examined the cleavage of PARP in the cells treated with M_4_N-based combination treatments. The data (Fig 2Aa) showed that overall a significantly greater PARP cleavage was observed for the cells treated with combination treatments than single drug treatments. In addition the data also showed that the combination treatment of M_4_N with Etoposide or UCN-01 yielded a greater amount of both the p89 and p24 fragments than that with Rapamycin. This data agreed with the caspase-7-related data (Fig 2A and 2B) indicating that caspase-7 activity was greater with combination treatment containing Etoposide or UCN-01 than that with Rapamycin.

The activation of the mitochondria-associated caspase-9/3-dependent cell death mechanism is usually associated with depolarization of mitochondrial membrane potential (ΔΨ_m_) [25]. For this reason we next conducted experiments using JC-1 dye to measure the ΔΨ_m_ (Fig 2C). The JC-1 dye experiments showed that UCN-01 treatment but not Etoposide or Rapamycin treatment induced depolarization of the ΔΨ_m_ in a short period of time. More importantly it also showed that M_4_N induced hyperpolarization of the ΔΨ_m_ and prevented depolarization of ΔΨ_m_ induced by UCN-01.

### M_4_N treatment down-regulates autophagy

**Fig 3:**
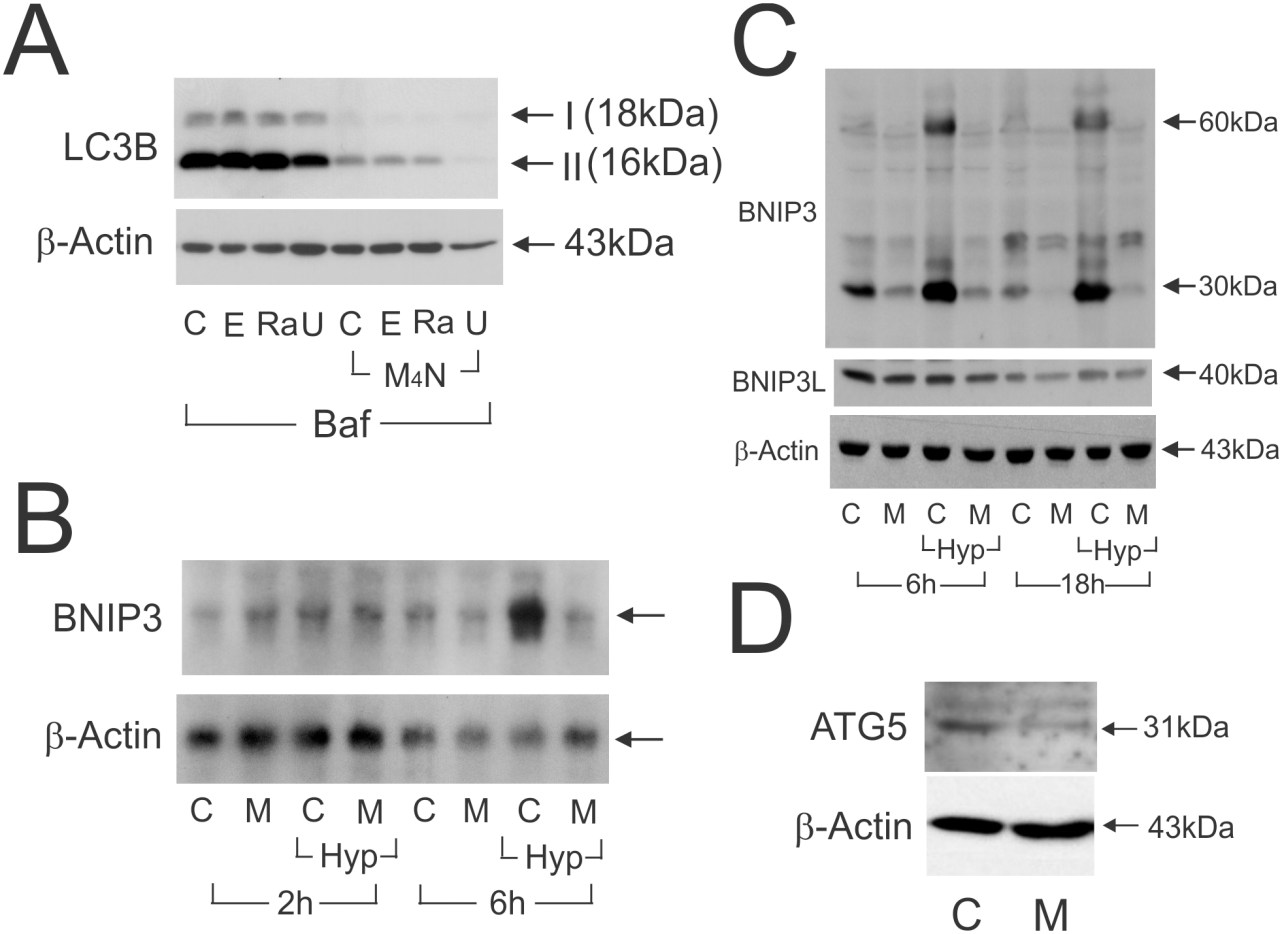
Suppressive effect of M_4_N on autophagy. **A:** Effect of M_4_N on autophagy in LNCaP cells. The expression of LC3B-I and II was examined by the western blotting in LNCaP cells treated with combination treatments of M_4_N with etoposide, rapamycin, or UCN-01 for 18 h in the presence of bafilomycin A_1_ (100 nM), an autophagosome degradation inhibitor. Bafilomycin A_1_ was added to measure the net activity of autophagy. The concentrations of M_4_N, etoposide, rapamycin, and UCN-01 were 80, 20, 20, and 5 μM, respectively. C: Control, E: etoposide, Ra: rapamycin, U: UCN-01. **B:** The mRNA expression of *BNIP3* gene, examined by northern blotting, in LNCaP cells treated with M_4_N (80 μM) under normoxic or hypoxic condition for 2 or 6 h. Hyp: hypoxic condition. **C:** The expression of BNIP3 and BNIP3L, examined by the western blotting, in LNCaP cells treated with M_4_N (80 μM) under normoxic or hypoxic condition for 6 or 18 h. **D:** The expression of ATG5, examined by the western blotting, in LNCaP cells treated with M_4_N (80 μM) for 18 h. β-Actin was used as a control.

Autophagy is a process that degrades damaged or unnecessary cellular components for the purpose of recycling them as resources, and is activated by nutrient deprivation conditions as a part of cellular defense mechanisms [26]. LC3B-II expression is widely known to correlate well with autophagic activity and used as an indicator for autophagy [27].western blot data (Fig 3A) showed that M_4_N suppressed LC3B-II net formation almost completely, indicating that M_4_N inhibited autophagy. Next we examined the effect of M_4_N on expression of BNIP3 by northern and the western blotting. BNIP3 is a BH3-only protein and is known to be involved in both apoptosis and autophagy [28–30]. Because BNIP3 is inducible by hypoxia, its expression was examined under both normoxic and hypoxic conditions. Treatment with M_4_N for 6 h effectively reduced both mRNA and protein expression of BNIP3 (Fig 3B and 3C), indicating that M_4_N suppressed *BNIP3* expression at the transcriptional level. The data also showed that M_4_N was able to suppress the basal as well as hypoxia-induced expression of *BNIP3* to almost null (Fig 3C). Additionally the Western blotting (Fig 3C) showed that M_4_N did not change the expression of BNIP3L, a protein related to BNIP3 [28]. The effect of M_4_N on the expression of ATG5, a key component for autophagy machinery [31], was then examined and western blot analysis (Fig 3D) showed that M_4_N significantly suppressed ATG5 expression in less than 18h. The data thus demonstrated that M_4_N suppressed autophagy by modulating multiple cellular components crucial for autophagic mechanisms such as ATG5 and BNIP3.

### M_4_N broadly modulates metabolic pathways

**Fig 4:**
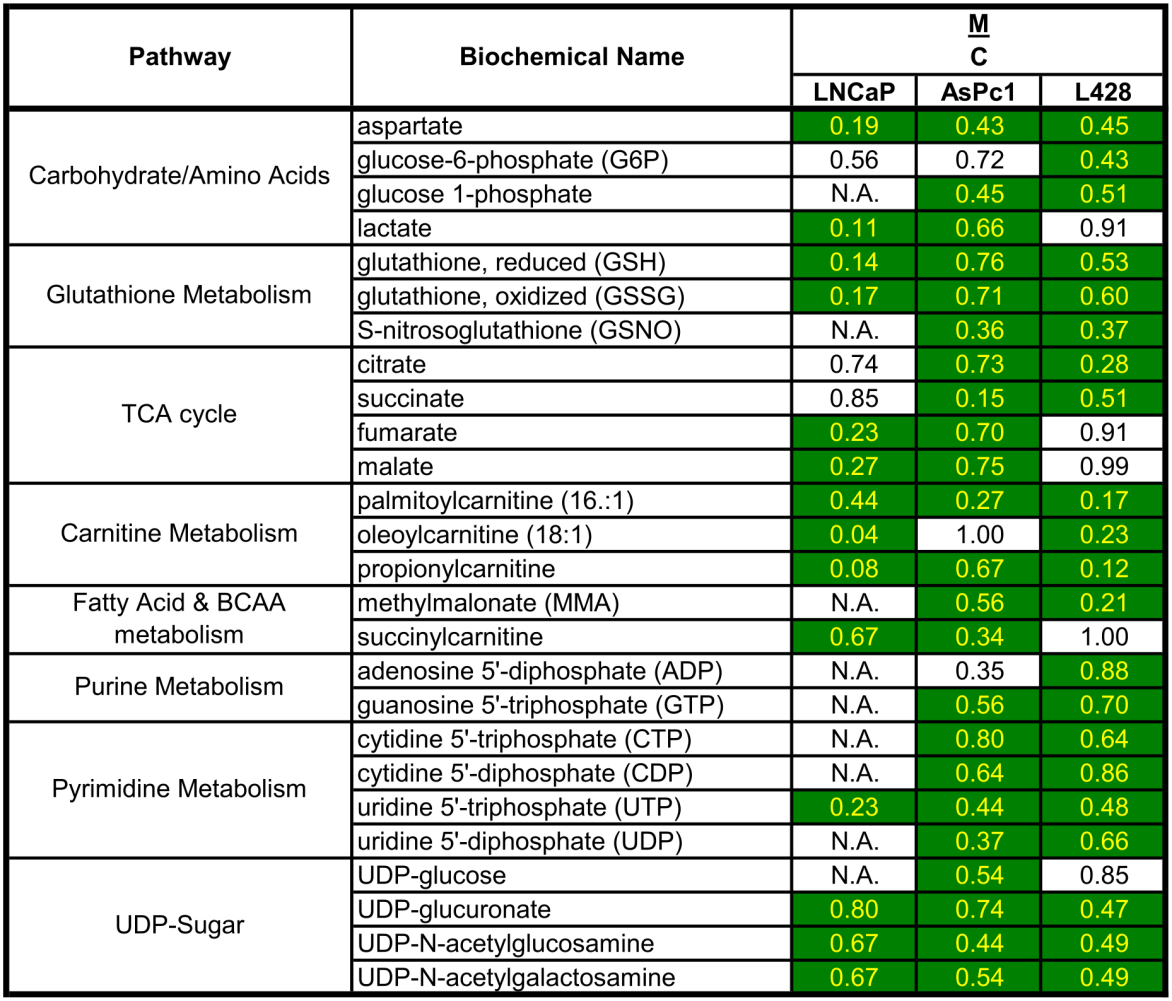
Metabolites whose cellular contents were suppressed by M_4_N treatment in common with three cell lines (LNCaP, AsPC-1, and L428). LNCaP, AsPC-1 and L428 cells were treated with M_4_N (80μM) for 8 h and metabolite contents of the samples were measured by LC/GC mass spectroscopy by Metabolon (S1 Table). Among the metabolites examined, only the metabolites whose contents were significantly (p≤0.05) suppressed by M_4_N in at least two out of three cell lines were selected and listed here under the additional condition that the effect of M_4_N on the contents of metabolites in the third cell line was a suppression, no significant change, or not examined. 'M/C' indicates the ratio of metabolite contents for the samples treated with M_4_N vs. control. N.A. indicates 'data not available'. The exceptions for these rules were ADP and UDP-glucose (whose contents were both suppressed by M_4_N in two of the three cell lines but the difference was statistically significant only in one of the two cell lines. In the third cell line the data was not available). The numbers for the ratio shaded in green indicate that metabolite contents were statistically smaller in the treated samples than the control while those without shades indicate that there was not a statistical difference between the control and the treated samples.

To understand the mechanism of synergy in the anticancer effect (Fig 1, S1 Fig), cellular contents of the biochemical metabolites and profiles for mRNA were examined searching for possible physiological changes following M_4_N treatment in LNCaP human prostatic cancer, AsPc-1 human pancreatic cancer, and L428 human Hodgkin lymphoma cells. First we examined the effect of M_4_N on metabolite contents in these cells (performed by Metabolon, Co. Ltd) [32]. The metabolite assay (S1 Table) showed broad influences of M_4_N on various principal metabolic pathways. Since the shortage of particular metabolites has more substantial impacts on cellular physiology than the abundance of them, we selected the metabolites whose cellular contents were consistently reduced by M_4_N in at least two out of three cell lines and unchange or undetermined in the third (Fig 4).

**Fig 5:**
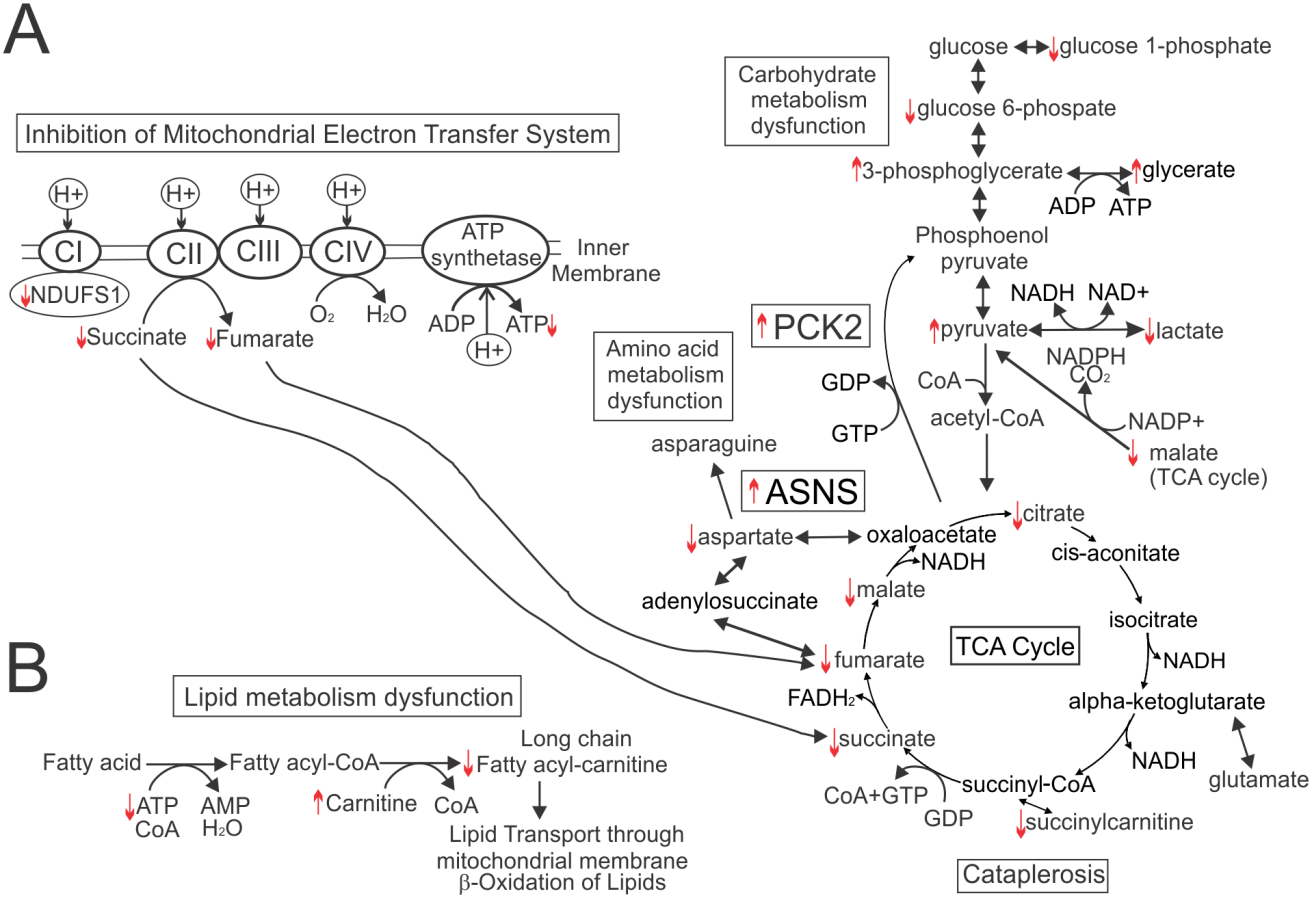
Effect of M_4_N on carbohydrate and lipid metabolisms in LNCaP, AsPC-1, and L428 cells. **A:** A schema for glycolysis-, TCA-cycle-, and mitochondrial electron transport system-related metabolic pathway. The raw data of the metabolite assay is shown in S1 Table. The selection of S1 Table can be found in Fig 4. The upward pointing arrows indicate that the contents of the metabolites associated with these arrows were significantly (p≤0.05) induced by M_4_N in at least two out of three cell lines (LNCaP, AsPC-1, and L428 cells) under the additional condition that the effect of M_4_N on the contents of these metabolites in the third cell line was an induction without statistical significant difference, no significant change, or not examined. Meanwhile the downward pointing arrows indicate that the contents of the metabolites associated with these arrows were significantly (p≤0.05) suppressed by M_4_N in at least two out of three cell lines (LNCaP, AsPC-1, and L428 cells) under the additional condition that the effect of M_4_N on the contents of these metabolites in the third cell line was a suppression without statistical significant difference, no significant change, or not examined. The exceptions for these rules are glucose 6-phosphate (whose content was suppressed by M_4_N in all three cell lines but the difference was statistically significant only in L428 cells), 3-phosphoglycerate (whose content was significantly induced by M_4_N in LNCaP but not in AsPC-1 and L428 cells) and pyruvate (whose content was significantly induced by M_4_N in AsPC-1 and L428 cells but significantly suppressed in LNCaP cells). 3-Phosphoglycerate and pyruvate are marked with associated upward pointing arrows because the data (S1 Table) indicated that M_4_N accumulated these metabolites and stalled glycolysis, although how M_4_N affected these metabolites was variable depending on the cell line (refer to the text). The effect of M_4_N on ATP content in the whole cells is shown in Fig 6A. The effect of M_4_N on NDUFS1 expression is shown in Fig 6C. The result indicating the increase in mRNA expression of phosphoenolpyruvate carboxykinase 2 (PCK2) and asparagine synthase (ASNS) by M_4_N (80 μM) treatment for 6 h was from deep RNA sequencing analysis shown in Table 1. **B:** A schema for lipid metabolic pathway. The meanings of the upward and downward pointing arrows in this schema are the same as those in the schema for glucolysis-, TCA-cycle-, and mitochondrial electron transport system-related metabolic pathway (Fig 5A). Long chain fatty acyl-carnitine includes palmitoylcarnitine (16:1) and oleoylcarnitine (18:1). When fatty acids are degraded through β-oxidation cycle, they need to be converted to acyl-carnitines to be transported inside the mitochondria. Thus the data indicated that M_4_N should suppress β-oxidation cycle by reducing production of long chain acyl-carnitines.

Many TCA cycle-related metabolites (citrate, succinate, fumarate, and malate) were depleted by M_4_N treatment (Fig 4). Only the content of α-ketoglutarate was increased slightly by M_4_N among the TCA-cycle-related metabolites (S1 Table). The effect of M_4_N on glucolysis/gluconeogenesis-related metabolites was variable depending on the cell line (S1 Table) although the content of glucose-6-phosphate and glucose-1-phosphate was consistently reduced by M_4_N treatment (Fig 4). Glucose-1-phosphate is converted to UDP-glucose by glucosyltransferase reactions with UTP. The content of both UDP-glucose and UTP was reduced by M_4_N as well (Fig 4), indicating that the metabolisms related to glucose-6-phosphate/glucose-1-phosphate/UDP-glucose were overall suppressed by M_4_N. The content of 3-phosphoglycerate, a key metabolite in the glucolysis/gluconeogenesis pathway, was significantly increased by M_4_N in LNCaP cells but not in AsPC-1 or L428 cells, while the content of pyruvate, which is also a key glycolysis metabolite, was significantly increased by M_4_N in AsPC-1 and L428 cells but not in LNCaP cells (S1 Table). This indicated that although the effect of M_4_N on glycolysis-related metabolites was variable depending on cell lines, M_4_N stalled glycolysis in all the three cell lines examined and accumulated some glycolysis-related metabolites such as 3-phosphoglycerate and pyruvate. Meanwhile the content of lactate was consistently reduced by M_4_N treatment (Fig 4), which indicated that the conversion from pyruvate to lactate was suppressed by M_4_N. In conclusion, the data related to carbohydrate metabolism (Fig 5A) overall showed that M_4_N reduced the content of TCA cycle-related metabolites while accumulating certain glycolysis-related metabolites, and thus induced cataplerosis (cataplerosis is defined here as a metabolic condition which occurs under the shortage of TCA cycle-related metabolites) [33].

The metabolite data also showed that M_4_N significantly reduced the content of aspartate in all three cell lines (Fig 4). Since aspartate is produced either from oxaloacetate by a transamination reaction or from fumarate through adenylosuccinate, M_4_N-mediated cataplerosis (Figs 4 and 5A) was a cause of reduced production of aspartate [33]. The metabolite data further showed that M_4_N modulated lipid metabolism as well. Although M_4_N differently modulated the contents of various types of lipids depending on cell lines (S1 Table), M_4_N consistently reduced the contents of long-chain acyl-carnitine such as palmitoylcarnitine and oleoylcarnitine (Fig 4) and increased the contents of carnitine and its derivatives in all three cell lines (S1 Table). Since long-chain acyl-carnitine is essential for transporting long-chain fatty acids throughthe mitochondrial membrane [34] the data indicated that M_4_N should reduce β-oxidation of fatty acids (Fig 5B).

### M_4_N suppresses energy metabolism

**Fig 6:**
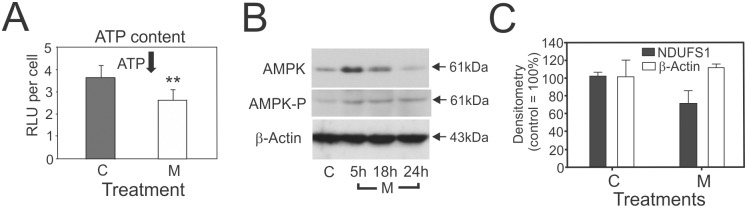
Effect of M_4_N on ATP. **A:** Effect of M_4_N on cellular ATP content in LNCaP cells. LNCaP cells were treated with M_4_N (80 μM) for 8 h. Data are presented as mean±SD from five samples. Two asterisks indicate that the difference between the control (C) and the M_4_N (M) treatment group was statistically significant by *t-*test at the error rate of less than 0.1%. **B:** Effect of M_4_N on the expression of AMPK. The expression of AMPK and AMPK-P (T174) was measured by the western blotting for LNCaP cells treated with M_4_N (80 μM) for 5, 18, or 24 h. β-Actin was used as the control. **C:** Effect of M_4_N on the expression of NDUFS1. The expression of NDUFS1 was measured by the western blotting for LNCaP cells treated with M_4_N (80 μM) for 5 h. The densitometry data from this raw data are presented as mean±SD from three independent samples. β-Actin was used as the control. The difference between the control and M_4_N-treated samples in NDUFS1 expression was statistically significant by *t*-test at the error rate of less than 1%.

**Fig 7:**
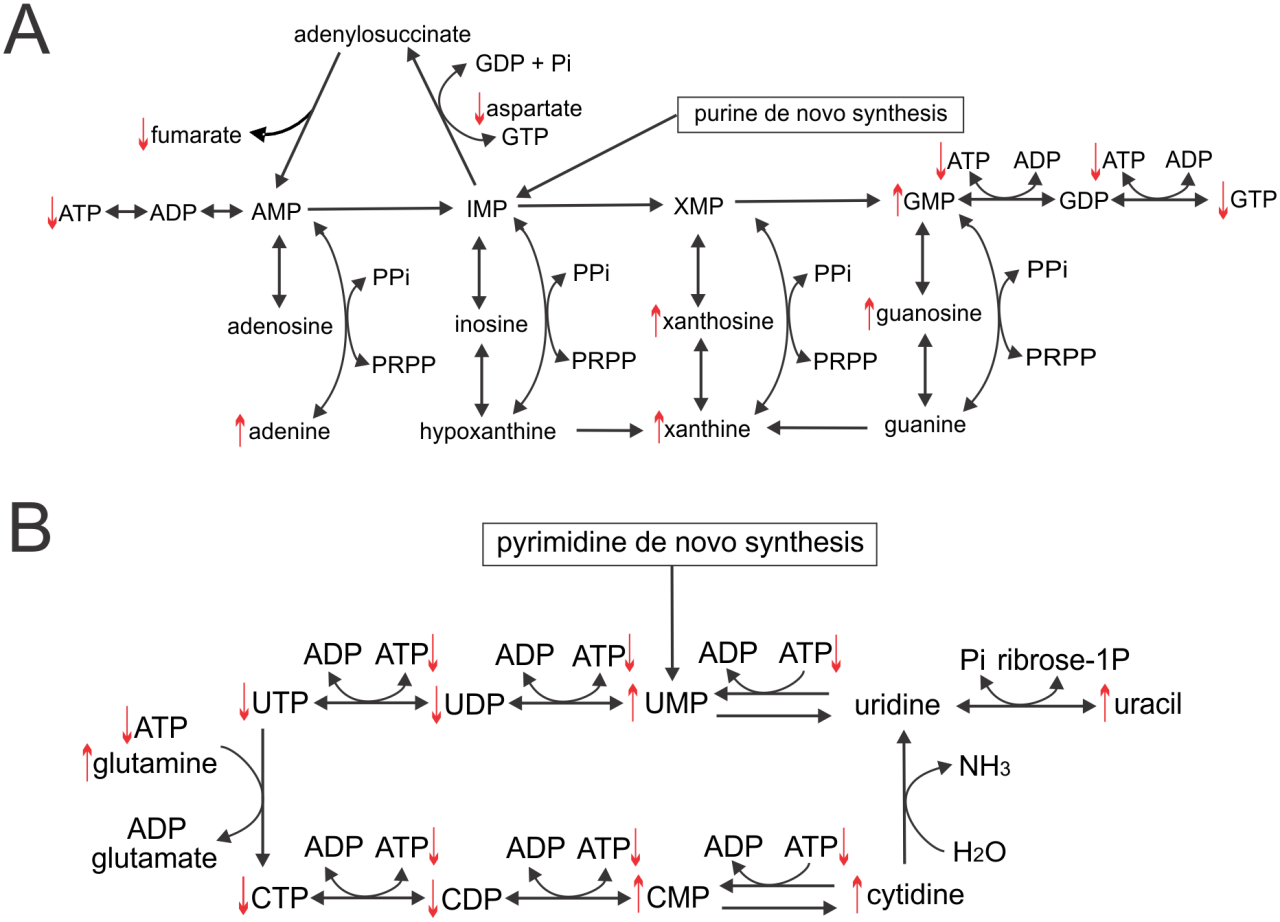
Effect of M_4_N on nucleic acid metabolism in LNCaP, AsPC-1, and L428 cells. Schemas for purine (**A**) and pyrimidine (**B**)-related metabolic pathways. The raw data of the metabolite assay is shown in S1 Table. The selection of S1 Table can be found in Fig 4. The upward pointing arrows indicate that the contents of the metabolites associated with these arrows were significantly (p≤0.05) induced by M_4_N in at least two out of three cell lines (LNCaP, AsPC-1, and L428 cells) under the additional condition that the effect of M_4_N on the contents of these metabolites in the third cell line was an induction without statistical significant difference, no significant change, or not examined. Meanwhile the downward pointing arrows indicate that the contents of the metabolites associated with these arrows were significantly (p≤0.05) suppressed by M_4_N in at least two out of three cell lines (LNCaP, AsPC-1, and L428 cells) under the additional condition that the effect of M_4_N on the contents of these metabolites in the third cell line was a suppression without statistical significant difference, no significant change, or not examined. The exception to these rules was xanthine (whose content was induced by M_4_N in all three cell lines but the difference was statistically significant only in L428 cells). The effect of M_4_N on ATP content in the whole cells is shown in Fig 6A. PRPP: phosphoribosyl pyrophosphate, PPi: pyrophosphate, Pi: phosphate.

Pardini *et al*. showed that NDGA, a precursor compound upon which M_4_N was based, inhibited the mitochondrial electron transport system [6–7], which suggesting that M_4_N might modulate energy metabolism (Figs 4 and 5, and S1 Table) through an effect on the mitochondria. To test this hypothesis, a luminescence-based enzymatic assay was conducted to measure the whole cell ATP content in LNCaP cells treated with M_4_N. The assay (Fig 6A) showed that M_4_N significantly reduced ATP content in 5 h. AMPK is known to function as a cell signaling sensor for ATP, ADP, and AMP and to be activated (phosphorylated) when the ratio of AMP/ATP content is high [35, 36]. The ratio of AMP/ATP was augmented by M_4_N treatment since AMP content was unchanged by M_4_N treatment according to the metabolite assay (S1 Table). As expected, western blot analysis showed that M_4_N induced the expression of both total AMPK and its phosphorylated form (T174) in 5 h (Fig 6B), indicating that AMPK was activated by M_4_N and the M_4_N-treated cells were starved for ATP. NADH dehydrogenase (ubiquinone) Fe-S protein 1 (NDUFS1) is a subunit of mitochondrial complex I. It has been shown that the activity of the electron transport system is suppressed when the gene coding for this protein is mutated [37]. We found that the expression of NDUFS1 was suppressed in LNCaP cells after 5h of M4N treatment(Fig 6C), indicating that M_4_N was able to interfere with certain components of electron transport system. Hyperpolarization of the ΔΨ_m_ is generally considered to be a condition associated with blockage in the electron transport system and low ATP generation [38]. Therefore the result of the JC-1 dye experiments described above (Fig 2C) as well as other mitochondria-related data described here (Fig 6) supported the aforementioned premise that M_4_N as well as NDGA had inhibitory effects on the mitochondrial electron transport system [6, 7], suppressing oxidative phosphorylation and reducing cellular ATP contents. Alternatively the decrease in ATP might be the result of an M_4_N-dependent decrease in glycolytic flux. Fig 5A summarized the effect of M_4_N on the TCA cycle/electron transport system-related metabolisms. Succinate dehydrogenase which converts succinate to fumarate in TCA cycle is also known as an important component of complex II of electron transport system. Interestingly the contents of both succinate and fumarate were significantly reduced by M_4_N (Fig 4), indicating that M_4_N suppressed metabolic activity of both the TCA cycle and electron transport system. Fig 7 summarized the effect of M_4_N on nucleic acid-related metabolisms, based on metabolite data (Fig 4, S1 Table) and ATP assay (Fig 6A). In addition to reduced amount of ATP (Fig 6A), the contents of nucleoside triphosphates such as CTP, GTP, and UTP were also decreased by M_4_N while those of precursor metabolites for nucleoside triphosphates such as CMP, GMP, UMP, adenine, guanosine, and cytidine were all accumulated after M_4_N treatment. This was another evidence indicating that M_4_N suppressed biological energy status (or ATP content) in cancer cells since the production of nucleoside triphosphates (GTP, CTP, or UTP) required ATP (Fig 6A).

**Fig 8:**
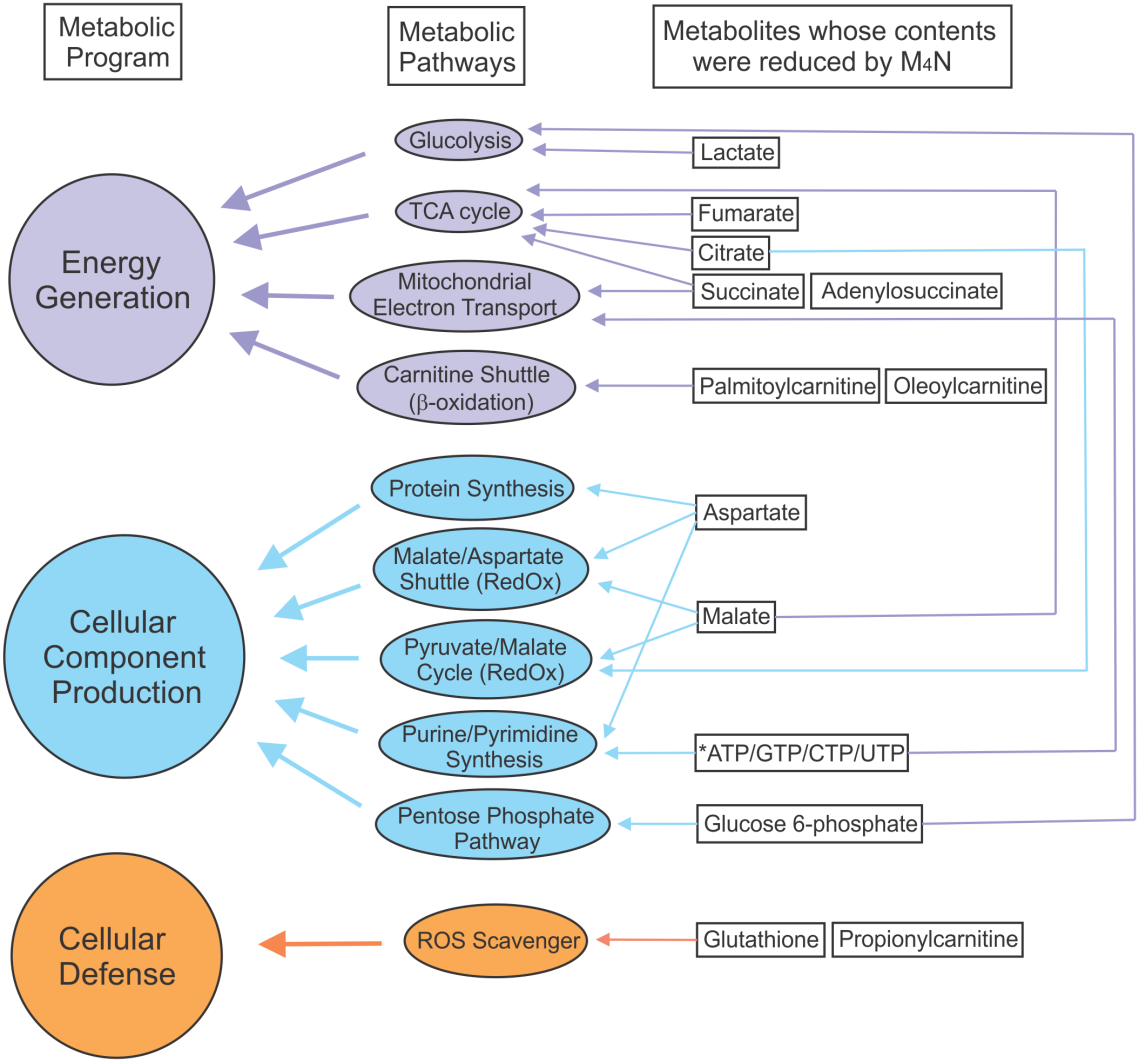
Analysis of the effect of M_4_N on metabolism. Fig 4 shows the metabolites whose contents were suppressed by M_4_N treatment in common with three cell lines (LNCaP, AsPC-1, and L428). The shortage of metabolites induced by M_4_N should negatively affect the metabolic pathways in which these metabolites were involved. Based on this premise, the data in Fig 4 was analyzed and this figure was drawn. On the right side, the names of metabolites from Fig 4 were shown in square boxes. In the middle, the metabolic pathways related to these metabolites were shown in elliptical circles. The relationships between metabolites and their related metabolic pathways were depicted by thin arrows. On the left side, the three major functions of these metabolic pathways were shown in circles. The relationships between metabolic pathways and their related major functions were depicted by thick arrows. An asterisk indicates that ATP concentration was measured by luminescent-based enzymatic assay (Fig 6A). The analysis showed that M_4_N suppressed various metabolic pathways in both energy generation and cellular component production. Additionally the analysis showed that M_4_N suppressed ROS scavenging mechanisms as well.

As shown in Fig 4, M_4_N negatively affected both anabolic (energy generation) and catabolic (cellular component production) pathways due to the shortage of various metabolites. Additionally, the amounts of glutathione and propionylcarnitine [39–41], the two major reactive oxygen species (ROS) scavengers, were also decreased by M_4_N treatments. Fig 8 illustrated how deficiencies of these metabolites, by affecting various metabolic pathways, should have made major impacts on the three cellular programs (energy generation, cellular component production, and cellular defense) of cancer cells. The fact that M_4_N alone was able to modulate various types of cellular metabolism in less than 8 h indicated that cellular metabolism was already greatly altered by M_4_N treatment long before the synergistic induction of cell death by combination treatments occurred.

### M_4_N modulates metabolism-related genes

The data so far showed that M_4_N suppressed energy metabolism in only a few hours after treatment with the drug. Such a significant physiological change must be accompanied with various changes in gene regulation. To understand these initial impacts of M_4_N on gene transcription, we examined mRNA profile for LNCaP, AsPC-1, and L428 cells after treatment with M_4_N for 6 h by the DNA microarray analysis. To eliminate the influence on the interpretation of the data by the gene expression changes which were cancer-type specific and relatively unimportant for explaining ubiquitously proven anticancer effect of M_4_N combination treatment (Fig 1, S1 Fig), we selected the genes whose expression was commonly modulated by M_4_N treatment in all three cell lines. Surprisingly this analysis showed that the expression of only sixteen genes were commonly modulated by M_4_N for these cell lines. A detailed analysis according to the general knowledge about these genes revealed that the functions of six out of these sixteen genes were predominantly related to metabolism regulation and stress transduction leading to ATF4/DDIT3/CHAC1 activation [42] (Table 1).

**Table 1:** Metabolism-related data from combined results of DNA microarray analysis about the effects of M_4_N treatment in three different cell lines (LNCaP, AsPc-1, and L428).

Group	Gene ID	Gene Name	log2 change		
			LNCaP	AsPc-1	L423
Metabolism-related	PCK2	phosphoenolpyruvate carboxykinase [GTP] (A mitochondrial enzyme that converts oxaloacetate to phosphoenolpyruvate with GTP)	1.39	1.85	2.97
	ASNS	asparagine synthetase (glutamine-hydrolyzing) (An amino acid depletion sensor; involved in GCN2 activation)	1.68	2.69	2.95
Stress-related (ATF4/DDIT3/CHAC1 pathway)	ATF3	activating transcription factor 3 (A CREB family transcription factor, related to stress)	4.51	4.59	2.46
	CEBPB	CCAAT/enhancer binding protein (C/EBP), beta (A transcription factor containing bZIP; related to stress)	2.71	2.00357	3.59
	CHAC1	ChaC, cation transport regulator-like 1 (Cation transporter; Also it degrades glutathione into 5-oxoproline and cysteinylglycine)	4.96	3.44	5.03
	SESN2	sestrin-2 (A gene inducible by metabolic stress; a cordinator of AKT1 and mTOR1 signals protect cell from stress via AMPK)	2.42	4.89	4.39

The genes whose expression was significantly (p≤0.05) modulated by M_4_N (80 μM) treatment for 6 h in common with all three cell lines (LNCaP, AsPc-1, and L428) were selected. There were only sixteen genes which were significantly modulated by M_4_N for all these cell lines. Among these genes, six genes which were involved in metabolism-related functions were listed in this table. The numbers in the column 'log2 change' indicate the ratio of mRNA expression of M_4_N-treated samples against the control by the log2 scale for each gene.

Table 1 showed that two out of the six genes (PCK2, ASNS) were involved in metabolism regulation [33, 43]. It was shown that transgenic mice overexpressed with PCK2 gene developed cataplerosis [33], which indicated that PCK2 induction mediated by M_4_N was involved in the mechanisms by which M_4_N-induced cataplerosis (Figs 4 and 5A). ASNS is known as an enzyme for converting aspartate to asparagine. Thus the induction of ASNS gene by M_4_N (Table 1) leads to reduction of aspartate induced by M_4_N (Fig 5). Meanwhile it was shown that the expression of three genes (ATF3, CHAC1, and CEBPB), which were important components of ATF4/DDIT3/CHAC1-related stress signal transduction mechanism, were augmented by M_4_N [42, 44, 45]. SESN2, whose expression was augmented by M_4_N as well, has functions related to both AMPK (Fig 6B) and stress-related signal transduction mechanism [46]. These stress-related genes are also related to metabolic regulation since metabolic stress is among the major stimulators for these genes.

### M_4_N induces oxidative stress

**Fig 9:**
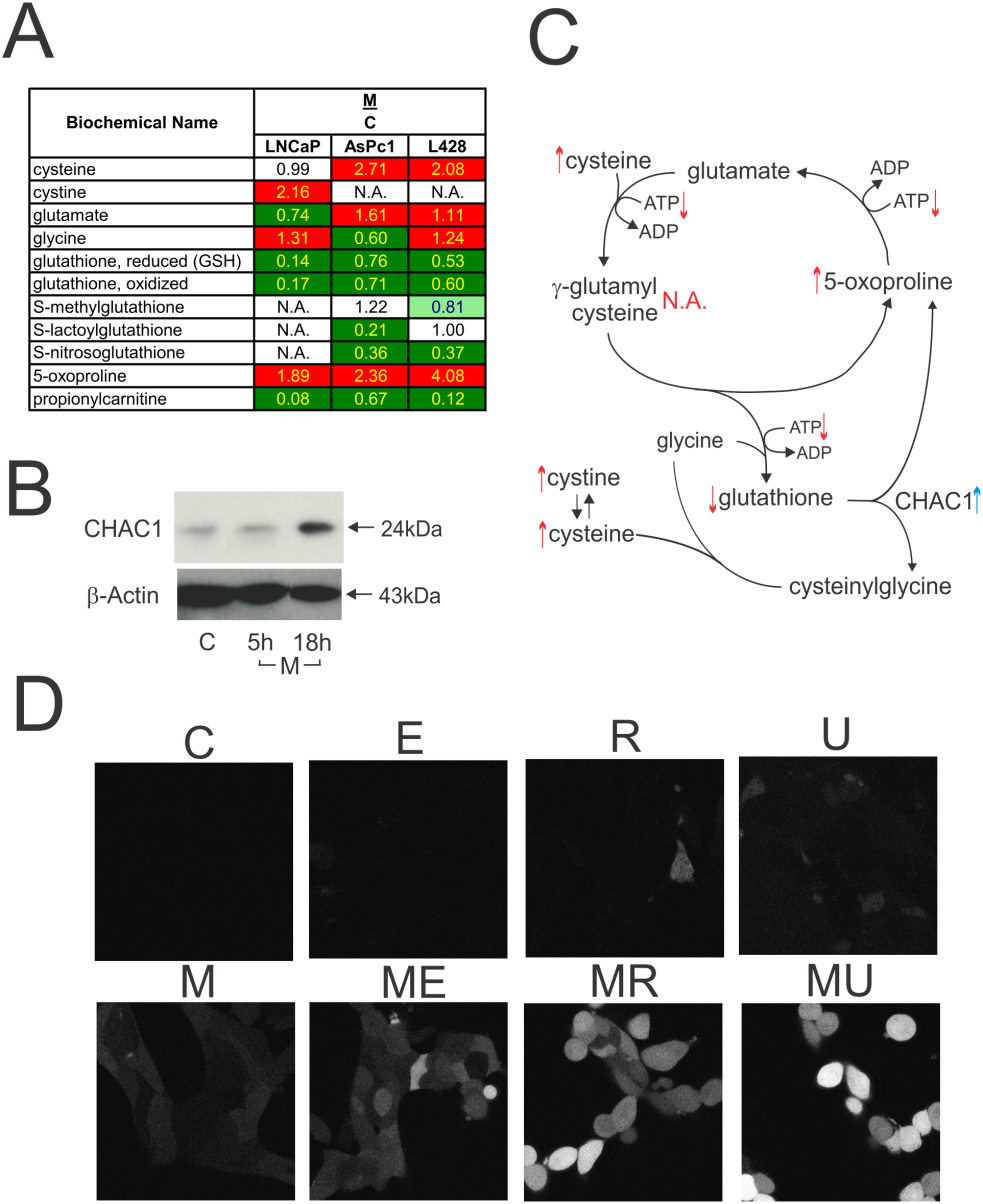
Effect of M_4_N on glutathione metabolism and ROS (reactive oxygen species) production. **A:** Effect of M_4_N on the contents of glutathione-related metabolites. This data is a selection from S1 Table. LNCaP, AsPC-1, and L428 cells were treated with M_4_N for 8 h and metabolite contents of the samples were measured by LC/GC mass spectroscopy by Metabolon. 'M/C' indicates the ratio of metabolite contents for the samples treated with M_4_N vs. the control. For paired comparisons between the control and M_4_N-treated samples, strongly shaded cells indicate statistically significant differences at p≤0.05 while lightly shaded cells indicate them at 0.05<p<0.10. Inside the table, red indicates that the mean values are significantly higher for that comparison while green values significantly lower. **B:** Effect of M_4_N on the expression of CHAC1. The LNCaP cells were treated with M_4_N (80 μM) for either 5 or 18 h and the expression of the protein was measured by the western blotting. β-Actin was used as the control. **C:** A schema for M_4_N-regulation of glutathione metabolism. The upward pointing arrows indicate that the contents of the metabolites associated with these arrows were significantly (p≤0.05) induced by M_4_N in at least two out of three cell lines (LNCaP, AsPC-1, and L428 cells) under the additional condition that the effect of M_4_N on the contents of these metabolites in the third cell line was an induction without statistical significant difference, no significant change, or not examined. Meanwhile the downward pointing arrows indicate that the contents of the metabolites associated with these arrows were significantly (p≤0.05) suppressed by M_4_N in at least two out of three cell lines (LNCaP, AsPC-1, and L428 cells) under the additional condition that the effect of M_4_N on the contents of these metabolites in the third cell line was a suppression without statistical significant difference, no significant change, or not examined. The exception to these rules was cystine (whose content was statistically significantly induced by M_4_N only in LNCaP cells. In other two cell lines the data are not available). The effect of M_4_N on ATP content in the whole cells is shown in Fig 6A. 'N.A.' indicates that the data on these particular metabolites are not available. The blue arrow indicates that CHAC1 expression was enhanced by M_4_N treatment (Fig 6B). The effect of M_4_N on ATP content in the whole cells is shown in Fig 6A. **D:** Effect of combination treatments on ROS production in LNCaP cancer cells. The cells were treated with M_4_N (80 μM), etoposide (30 μM), rapamycin (30 μM), or UCN-01 (2 μM). ROS content was measured at 5 h after treatment. C: control, E: etoposide, R: rapamycin, U: UCN-01, M: M_4_N, ME: M_4_N+etoposide, MR: M_4_N+rapamycin, MU: M_4_N+UCN-01.

The metabolite assay showed that M_4_N significantly depleted glutathione (both reduced and oxidized), a primary antioxidant, and its various derivatives while significantly increasing the amount of degradation products from glutathione such as 5-oxoproline, cysteine, and cystine (Figs 4 and 9A). As described above, it was shown that M_4_N significantly induced *CHAC*1 expression (Table 1, Fig 9B), which implied a possible involvement of CHAC1 in M_4_N-mediated reduction of cellular glutathione content, since this enzyme had activity to degrade glutathione into 5-oxoproline, cysteine (cystine), and glycine [39, 40]. Additionally M_4_N depleted propionylcarnitine, another superoxide scavenger [41] (Fig 4). This indicated that M_4_N decreased the cellular content of multiple anti-oxidants. The effect of M_4_N on glutathione-related metabolisms is summarized in Fig 9C.

The accumulative data demonstrates that M_4_N has various activities including blockage of the mitochondrial electron transport system, autophagy suppression, depletion of anti-oxidants, and modulations of acyl-carnitine metabolism. All these activities have been shown to be involved in oxidative stress induction accompanied with ROS production [47–50]. The fluorescent dye-based assay (Fig 9D) demonstrated that M_4_N induced ROS production in LNCaP cells and more importantly that M_4_N markedly increased ROS production when they were treated in combination with either etoposide, rapamycin, or UCN-01.

## Discussion

Since Warburg first described that cancer cells utilize bioenergetic metabolic pathways differently from normal cells [51], there have been various efforts to take advantage of this difference to develop anticancer therapies [52–55]. The metabolite analysis (Figs 4–8, S1 Table) showed that M_4_N extensively modulated energy metabolism, leading to cataplerosis induction, disruption of amino acid-related metabolism with aspartate depletion, modulation of lipid metabolism with long chain acyl-carnitine depletion, and depletion of nucloside triphosphates. In addition several lines of evidence indicated that M_4_N suppressed mitochondrial electron transport system (Figs 2C and 6). The TCA cycle and mitochondrial electron transport system are intricately related (Fig 5A). For instance succinate dehydrogenase is a major component of complex II in the mitochondrial electron transport system as well as a TCA cycle enzyme [56]. Thus blockage of electron transfer in the mitochondria should result in low metabolic activity of TCA cycle. This suggests that cataplerosis induced by M_4_N might be related to suppression of the electron transport system activity induced by M_4_N. It was The findings reported here are supported by a previous report that NDGA (a precursor of M_4_N) suppressed succinate dehydrogenase enzyme activity [7] (Figs 4 and 5A). *PCK2* was one of the eight metabolism-related genes selected by the deep RNA sequencing analysis (Table 1). It was shown that transgenic mice overexpressed with the *PCK2* gene developed cataplerosis [33], suggesting that *PCK2* induction mediated by M_4_N may be involved in the mechanisms by which M_4_N induced cataplerosis (Figs 4 and 5A). Imbalance of energetic flow due to cataplerosis should have strong impacts on cancer cells since cancer cells proliferate rapidly, so that they require a great deal of new materials and energy to sustain their growth. For instance, it was shown that due to cataplerosis, malate was depleted in M_4_N-treated cells (Figs 4 and 5A), leading to the low NADPH availability, since one of the main sources for NADPH is the reaction catalyzed by malic enzyme which converts malate to pyruvate (Figs 5A and 8; Pyruvate/Malate cycle). NADPH is essential for anabolic reactions which are required for building materials during cell growth. Thus even a depletion of malate alone, which occurs under the influence of M_4_N treatment, could induce negative impacts on tumor growth.

It was shown that M_4_N treatment reduced the content of aspartate in all three cell lines examined (Fig 4). Aspartate is derived from oxaloacetate and fumarate, both TCA-cycle metabolites (Fig 5A). This indicated that M_4_N treatment resulted in aspartate depletion due to its effect to induce cataplerosis. Additionally the deep RNA sequencing data showing that the asparagine synthetase (*ASNS*) gene [43] was induced by M_4_N (Table 1), indicated that *ASNS* induction was also a probable cause of aspartate reduction in the presence of M_4_N, since the ASNS enzyme converts aspartate to asparagine. Meanwhile, in all three cell lines examined, it was shown that M_4_N consistently reduced long-chain acyl-carnitine contents (Figs 4 and 5B), indicating that lipid β-oxidation cycle was suppressed by M_4_N, since acyl-carnitines are required for initiation of this cycle. The conversion from acyl-CoA to acyl-carnitine is catalyzed by two mitochondria membrane-bound enzymes called carnitine palmitoyltransferase (CPT) I and II. It was shown that an inhibition of CPT I decreased β-oxidation and induced antitumor effect in certain tumor cells [57], which suggested that anticancer activity of M_4_N might be partially derived from its effect to reduce acyl-carnitine contents in cancer cells. Fig 7 showed that M_4_N reduced the contents of ATP, CTP, GTP, and UTP, indicating that M_4_N had negative impacts on DNA and RNA generation.

The metabolites-related data in this study indicates that M_4_N has negative impacts on a wide range of metabolic pathways in both catabolism and anabolism (Fig 8). Recently it was reported that liver-X-receptors (LXR) reverse agonist SR9243 inhibited the Warburg effect and lipogenesis, and induced an anticancer effect [58], suggesting some similarity between M_4_N and SR9243 in their pharmacological actions. M_4_N inherently should have much broader molecular targets than SR9243 because M_4_N is a competitive inhibitor for a general gene transcription factor SP1 while SR9243 is a specific agonist for a nuclear receptor LXR. Our study showed that M_4_N also had many other pharmacological activities than its activity on metabolisms (Figs 2–9). Either way the studies on SR9243 and M_4_N indicate that the development of drugs with broad molecular targets could be an appropriate approach to design effective anticancer drugs.

To identify the exact targets of M_4_N in the mitochondria is out of the scope of this study, although there are several potential candidates. NDUFS1 and any mitochondrial proteins proven to be affected by NDGA including succinate dehydrogenase are among them (Fig 6C) [6–7, 37]. Another potential candidate is BNIP3. As shown in Fig 3B and 3C, M_4_N almost completely blocked the expression of BNIP3 protein in a fairly short period of time. There are a number of SP1 consensus sequences in the *BNIP3* gene promoter [59], indicating that M_4_N possibly inhibited transcription of BNIP3 mRNA by competitively inhibiting SP1 binding (Fig 3B). Recent studies showed that a basal activity of BNIP3 and its downstream target, BAX/BAK, played important roles in maintaining various mitochondrial functions [60, 61]. Glick et al. showed that there were major morphological and biochemical differences in the mitochondria from the liver of BNIP3 null mice and the control [60]. In regard to energy metabolism, there was significant similarity between their results and ours. For example, oxidative phosphorylation and lipid β-oxidation were suppressed in both cases. Additionally, Boohaker *et al*. showed that the basal expression of BAX, a downstream target of BNIP3, was required for mitochondrial respiration [61]. These studies on BNIP3 and BAX seem to indicate that M_4_N might be able to induce metabolic modulations in the mitochondria through its effect on BNIP3 and its downstream target BAX.

Under these metabolic conditions it is expected that autophagy be activated as it is an important mechanism to recycle unnecessary or damaged cellular components and to protect cells under nutrient deficient conditions [26]. However, it was found that M_4_N suppressed autophagy (Fig 3A). The suppressive effect of M_4_N on autophagy would be expected to aggravate the already poor metabolic conditions that M_4_N incurred on the cancer cells. The precise mechanism of autophagy inhibition by M_4_N is not totally clear. However, the suppressive effect of M_4_N on the expression of BNIP3 and ATG5 certainly plays important roles in this inhibitory effect, considering the crucial roles that these proteins play in the mechanism of autophagy (Fig 3B and 3D).

Deep RNA sequencing showed that there were sixteen genes (PCK2, ASNS, ATF3, CEBPB, CHAC1, SESN2, GTF2I, DUSP1, NGFR, TRIB3, TSC22D3, GADD45A, PPP1R15A, DDIT4, CTH, and SLC9A1) which were modulated by M_4_N in all three cell lines, LNCaP, AsPC-1, and L428 (M_4_N downregulated GTF2I and upregulated all the other genes). Table 1 shows the six genes of this set that are related to metabolic regulation. The roles of PCK2 in cataplerosis induction [33] and ASNS in aspartate metabolism were discussed above [43] (Fig 5A). Induction of ATF4/DDIT3/CHAC1 stress pathway-related genes (*ATF3*, *CEBPB*, and *CHAC1*) by M_4_N treatment was particularly of interest, since there was copious evidence that M_4_N exerted metabolic and oxidative stresses on the cells (Figs 3–9). The activation of the stress-related ATF4/DDIT3/CHAC1 pathway ultimately results in CHAC1 induction (Table 1, Fig 9B). CHAC1 is involved in both glutathione metabolism and calcium-related signal transduction [40, 62] and has been shown to play important roles in cell death mechanisms [42, 62]. This suggests that the stress pathway leading to CHAC1 induction played a key role in the anticancer efficacy M_4_N in the combination treatments. SESN2 functions to transduce biological stress to AMPK [46]. Its induction by M_4_N (Table 1) therefore likely played a role in M_4_N-mediated AMPK activation (Fig 6B), along with the aforementioned suppression of ATP cellular content (Fig 6A). Thus, overall the deep RNA sequencing data for six metabolism-related genes supported the metabolome data. Future investigation of the remaining ten genes should provide more insight into the mechanisms of pharmacological actions of M_4_N.

This study has revealed a number of previously undiscovered activities of M_4_N, including blockage of the mitochondrial electron transport system (Figs 5A and 6), autophagy suppression (Fig 3A), depletion of anti-oxidants (Figs 4, 9A and 9C), and depletion of long chain fatty acyl-carnitine (Figs 4 and 5B). All these activities have been shown to be involved in oxidative stress induction accompanied with ROS production [47–50]. In fact our studies showed that M_4_N and its combination treatments induced ROS production in a fairly short period of time after treatment (Fig 9D). Considering the enzymatic actions of CHAC1 in glutathione metabolism [40] (Fig 9C) and the importance of glutathione as a scavenger for ROS, CHAC1 probably plays some role in induction of ROS production induced by M_4_N and its combination treatments (Fig 9D). However, since the induction of CHAC1 takes some time after M_4_N treatment (Fig 9B), a direct effect of M_4_N on the mitochondria such as inhibition of electron transport system (Fig 6) probably plays a more significant role than CHAC1 in rapid (5h) ROS induction by M_4_N. ROS has diverse activities that include functioning as a carcinogen, a key actor of nonspecific immune system, and a component of certain signal transduction pathways [63]. However, when cellular contents of ROS reach a very high level, ROS starts imposing detrimental cytotoxic effect on the cells. Various anticancer therapies including radiotherapy and photodynamic therapy utilize this activity of ROS to eliminate cancer cells [63]. It was also shown that certain compounds which had activity to eliminate ROS scavengers significantly facilitated efficacy of anticancer drugs against melanoma [64]. Our study showed that although M_4_N treatment alone induced only modest increase in ROS generation, the combination treatment of M_4_N with second drugs greatly increased ROS generation (Fig 9D). This data strongly indicated that ROS generation was involved in an enhanced anticancer activity of M_4_N combination treatments.

The amount of active caspase-7 increased in all combination treatments, whereas active caspase-9 and -3 stayed at the same level or even decreased in some cases (Fig 2Aa, 2Ab and 2B). Recent studies showed that the principal function of caspase-7 is to cleave and inactivate poly-ADP ribose polymerase (PARP) and the HSP90 cochaperone p23 [24, 65]. Both proteins have important roles in DNA repair. In fact it was found that PARP cleavage (inactivation) was markedly increased by M_4_N combination treatments (Fig 2Aa), which suggested that DNA repair function might be compromised in M_4_N-treated cells. It was shown that PARP inhibitors increased efficacy of certain anticancer drugs [66]. This suggests that the ability of M_4_N to improve anticancer efficacy in combination treatments may partially rely on its activity to inactivate PARP. The combination treatment yielded two different sized caspase-7 fragments, p30 and p17 (Fig 2Aa). Previously it was shown that calpain was capable of cleaving caspase-7 in a very distinct manner [67, 68], generating a p30 fragment (enzymatically inactive) and a p17/18 fragment (enzymatically active). This indicates that other proteases like calpain may also be involved in caspase-7 cleavage following M_4_N combination treatments. The cell death induced by M_4_N combination treatments did not require caspase-3 activation (Fig 2Aa), which indicate that the combination treatments induce cell death by necrosis rather than by apoptosis. Additionally, cell death induced by the combination treatments included non-TUNEL positive yet Trypan blue exclusion assay-positive cell death (Fig 1B), providing evidence that non-apopotic cell death also played an important role

**Fig 10:**
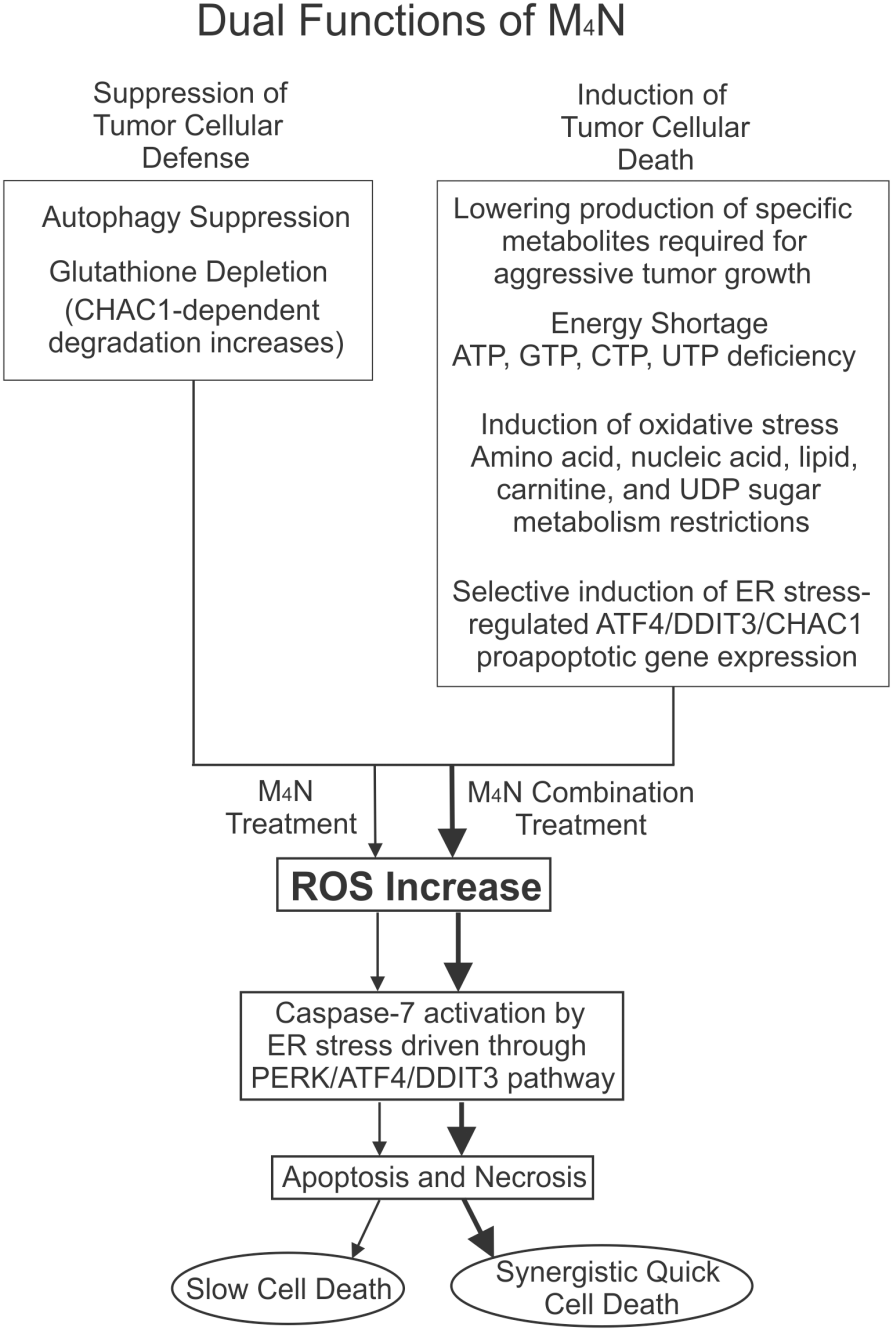
A schema depicting the mechanisms about how M_4_N combination treatments work. Processes depicted by thick arrows (in the combination treatment) operate better than those by thin arrows (in M_4_N treatment only).

The cytotoxic insults to cancer cells induced by anticancer drugs are countered by protective responses from the cancers [69, 70]. These protective responses quite often reduce the efficacy of the drugs. Thus a reasonable strategy is to the anticancer drugs with another drug which can reduce the cellular defense system of the cancer cells. Since autophagy is one of the principal protective cellular mechanisms, there have been numerous attempts to combine commercially available anticancer drugs with inhibitors of autophagy such as chloroquine [71]. Our study showed that M_4_N induced stress (oxidative and ATF4/DDIT3/CHAC1-related) by modulating various metabolic activities (mitochondrial electron transport system blockage along with cataplerosis and modulations of amino acid and lipid metabolism) in cancer cells, and also reduced cellular defense mechanisms (depletion of anti-oxidants and autophagy suppression) in cancers (Fig 10). Due to this dual action, M_4_N treatment induces stress in cancer cells and weaken them considerably. Although M_4_N by itself does not have strong cell death-inducing activities, it can synergistically act with a second anticancer drug to induce cell death by preconditioning the cancer cells with its dual action. The biochemical and physiological effects of M_4_N on cancer cells are quite extensive (Figs 2–9). Probably this is the reason why M_4_N is able to synergistically induce cell death with multiple anticancer drugs (Fig 1, S1 Fig) without a preferences for a specific class of drug (Fig 1, S1 Fig). Because of this unique property, Notwithstanding this broad range of activity,M_4_N, is very non-toxic, which will make the clinical application of this drug in multidrug therapy very feasible. Overall this quite impressive anticancer effect of M_4_N-based combination treatments suggest that this dual action might be one of the keys for developing effective anticancer chemotherapy.

## Materials and Methods

### Cell culture

Three prostate cancer (LNCaP, DU145, and PC3), three breast cancer (MCF-7, MDA-MB-231, and MDA-MB-468), two hepatoma (HepG2 and Hep3B), HT29 colon cancer, AsPC-1 pancreas cancer, K562 myelogenous leukemia, and LN229 glioblastoma cancer cell lines were purchased from American Type Culture Collection (Manassas, VA).The OC24 ascites ovarian cancer cell line was obtained from Dr. Ie-Ming, Shih (Johns Hopkins Medical Institutes, Baltimore, MD) [72]. The L428 Hodgkin lymphoma cell line was purchased from Leibniz Institute DSMZ-German Collection of Microorganisms and Cell Cultures (Germany). CAK24, Panc1.98, and Panc215 pancreatic cancer cell lines were obtained from Dr. James R. Eshleman. (Johns Hopkins Medical Institutes, Baltimore, MD) [73]. All of these cell lines are of human origin. LNCaP cells were cultured in RPMI1640 medium supplemented with glucose (14 mM), pyruvate (1 mM), and fetal bovine serum (FBS) (10%). DU145 cells were cultured in MEM supplemented with non-essential amino acids, glucose (21 mM), pyruvate (1 mM), and FBS (10%). PC3 cells were cultured in F-12K medium supplemented with FBS (10%). MCF-7 cells were cultured in DMEM supplemented with FBS (10%). MDA-MB-231 and MDA-MB-468 cells were cultured in Leibovitz’s C-15 medium supplemented with FBS (10%). HepG2 and Hep3B cells were cultured in MEM supplemented with FBS (10%). HT29 cells were cultured in McCoy’s 5A medium supplemented with FBS (10%). K562 and L428 cells were cultured in RPMI1640 medium supplemented with FBS (10%). Pancreatic cancer cell lines, AsPC-1, CAK24, Panc1.98, and Panc215, were cultured in DMEM supplemented with FBS (10%). LN229 cells were cultured in RPMI1640 medium supplemented with FBS (5%). OC24 cells were cultured in RPMI1640 medium supplemented with FBS (10%). All the tissue culture media contained penicillin (100 units/ml) and streptomycin (100 μg/ml). M_4_N stock solution were made in 100% dimethyl sulfoxide (DMSO). Final concentration of DMSO in the culture medium was 1.0%. When the cells were indicated to be cultured under hypoxic conditions, the cells were incubated at the oxygen concentration of 0.5% in a hypoxic chamber (BioSpherix Ltd, Lacona, NY).

### Reagents

Terameprocol (M_4_N) (10 mg/ml in CPE 25/30 formulation) was supplied by Erimos Pharmaceutical, LLC (Raleigh, NC), according to the method described [70]. Etoposide, rapamycin, UCN-01, and bafilomycin A_1_ were all from Sigma-Aldrich (St. Louis, MO). Anti-BNIP3 mouse monoclonal antibody was from Abcam (Cambridge, MA). Anti-caspase-9 (full and cleaved), anti-caspase-7 (full and cleaved p30 fragment), anti-caspase-7 antibody specific for p17 fragment, anti-cleaved caspase-3, anti-AMPKα, anti-phospho-AMPKα (Thr172), and anti-LC3B rabbit polyclonal antibodies were all from Cell Signaling Technology (Danvers, MA). Anti-PARP monoclonal antibody was from BD Pharmingen (San Jose, CA). Anti-BNIP3L polyclonal antibody was from Exalpha Biologicals (Watertown, MA). Anti-CHAC1 polyclonal antibody were from Santa Cruz Biotechnology (Santa Cruz, CA). Anti-ATG5 polyclonal antibody was from Abgent (San Diego, CA). Anti-NDUFS1 antibody was from Novus Biologicals (Littleton, CO). Anti-β-actin monoclonal antibody was from Sigma.

### Drug treatments for animals

T-cell-deficient male nude mice (nu/nu) were obtained from Charles River Laboratories (Wilmington, MA). Etoposide (4 mg/ml), rapamycin (3.75 mg/ml), and M_4_N (terameprocol, 10 mg/ml) were formulated separately in CPE 25/30 vehicle [74] and used independently without being mixed with other drugs. For drug combinations (M_4_N/etoposide, and M_4_N/rapamycin), etoposide, or rapamycin powder was further added to M_4_N in CPE 25/30 to make a final concentration of M_4_N (10 mg/ml) with etoposide (4 mg/ml), or rapamycin (3.75 mg/ml) respectively, in CPE 25/30 formulation. These drugs were intravenously injected into the tail vein of mice at the daily dose of 0.1 ml per mouse. Therefore, dosages of each injection were 1 mg/injection for M_4_N, 0.4 mg/injection for Etoposide, and 0.375 mg/injection for rapamycin. The drug injections were performed once every day, from day 3 until day 31 after tumor inoculation. The drugs were then injected once a week. The numbers of mice in each experimental group were 18, 5, 4, 4, 9 and 5 for the control, etoposide alone, rapamycin alone, M_4_N alone, M_4_N+etoposide, and M_4_N+rapamycin group, respectively. Protocols used in this study were approved by the Institutional Animal Care and Use Committee at the Johns Hopkins University (Baltimore, MD).

### Surgical orthotopic implantation of LNCaP tumors

LNCaP cells growing subconfluently were collected into cell culture medium without FBS and antibiotics. The cell concentration was adjusted with the same medium. After 50 μl of the medium containing about 2 × 10^6^ cells had been mixed with the same volume of Matrigel (BD Science, Bedford, MA), the combined solution was injected under the skin of nude mice. The tumor tissue growing subcutaneously was used for surgical orthotopic implantation. The operation was performed according to the method described by Wang *et al*. [14]. The tumor tissue extracted from the skin was excised into pieces of about 2-mm diameter. After the nude mice were anesthetized by 2,2,2-tribromoethanol (Aldrich Chemical Co. Inc., Milwaukee, WI), a small incision was made at the abdomen of each mouse and a tumor tissue piece was implanted near the prostate of each mouse. Three days after the operation, the intravenous injection of drugs was started.

### Computer analysis of the synergism between drugs

The synergism of combination drug treatments was analyzed by the Combosyn software (Combosyn Inc., Paramus, NJ), according to the methodology described by Chou and Talalay [20].

### Cell death assay

Terminal deoxynucleotidyl transferase dUTP nick end labeling (TUNEL) assay was conducted by using TUNEL apoptosis detection kits (Upstate, Temecula, CA), with some modifications. The cells were cultured in 12-well microwell culture dishes (Corning Inc., Corning, NY). After the treatment, both the cells floating in the tissue culture medium and those attached to the bottoms of wells were collected together into plastic tubes. After the cells were spun down at 700rpm, they were fixed with 10% formaldehyde in PBS(-) (phosphate buffered saline without calcium and magnesium) for 5 min and stored in PBS(-). The fixed cell samples were put on glass slides and dried in the air. The slides were first incubated in the solution containing 0.05% Tween-20, 0.2% BSA in PBS(-) for 15 min at room temperature. The samples were then treated with terminal deoxytransferase and biotin-dUTP included in the TUNEL assay kit for 60 min at room temperature, according to the manufacturer’s protocol. After the incubation, the samples were incubated with avidin-biotin complex (ABC reagent, Vector Laboratory Inc., Burlingame, CA) for 30 min at room temperature. After extensive washing with PBS(-), the DNA terminal ends of the samples were exposed by the peroxidase reaction using DAB as a substrate (peroxidase substrate kit, Vector Laboratory Inc.). The samples were counterstained by methyl green and embedded in VectaMount (Vector Laboratory Inc.). For the Trypan blue exclusion assay, the cells were washed with PBS(-) once and resuspended in PBS(-). One part of the resuspended cell solution was mixed with one part of 0.4% Trypan blue solution (Sigma). After 15 min, the numbers of cells with and without staining were counted. The percentage of stained cells to the total cell number (i.e., with + without staining) was calculated.

### Analysis of cell metabolites

LNCaP, AsPC-1, and L428 cells were treated with M_4_N (80 μM) for 8 h and quickly washed with PBS(-), and then immediately frozen with dry ice/ethanol. The samples were analyzed by Metabolon Inc. (Durham, NC) [31]. Alternatively LNCaP cells were treated with etoposide (20 μM) and their metabolites were analyzed in the same way by Metabolon Inc. to show the specificity of the effect of M_4_N.

### Deep RNA sequencing analysis

LNCaP, AsPC-1, and L428 cells were cultured in T75 flasks and treated with M_4_N (80 μM) for 6 h. RNA was extracted from the cells by Trizol reagent (Invitrogen, Carlsbad, CA) and was cleaned by RNeasy kit (Qiagen, Valencia, CA), according to the manufacturer’s protocol. The deep RNA sequencing analysis was done at the Deep Sequencing and Microarray Core, Johns Hopkins Medical Institutes (Baltimore, MD). To analyze RNA sequence data, reads were first mapped to human genome (hg19) using TopHat 1.4 [75] and differential expression was detected using the cuffdiff module in Cufflinks [76] and ensembl transcriptome as a guide.

### Measurement of ATP content in whole cells

LNCaP cells were seeded in black 96-well flat-bottom plates at a density of 1 × 10^4^ cells per well. ATP levels were quantified using the ATPLite 1-Step assay kit (PerkinElmer, Waltham, MA), according to the manufacturers’ protocol. Luciferase activity, reported as relative luminescence units (RLU), was measured on a Cary Eclipse fluorescence spectrophotometer (Varian, Palo Alto, CA). The luminescence was normalized to the number of cells per well. A standard curve of known ATP concentrations was also established to ensure that the experimental values were within an accurate range.

### Measurement of mitochondrial membrane potential (ΔΨm)

The cells were cultured in 6-well microwell dishes and treated with cell culture medium containing JC-1 dye (Cayman Chemical, Ann Arbor, MI) for 30 min, according to the manufacturer’s protocol. After the cells had been washed carefully, they were treated with M_4_N for 5 h. The cells were observed through a B29/Zeiss LSM 510 META laser confocal microscope (Carl Zeiss, Jena, Germany). The cell images were captured by two excitation lights, with 488-nm argon-ion and 568-nm argon-krypton lasers. Both JC-1 monomer and J-aggregates are detected by 488-nm excitation light, whereas only J-aggregates are detected by 568-nm excitation light. The ratio of the intensity of the emission light excited by 568-nm light to that excited by 488-nm light in every pixel of an image (the ratio is correlated with the ΔΨ_m_) was calculated by the imaging software (Carl Zeiss). The ratio is correlated with the ΔΨ_m_. In the figure, the ratio is shown by pseudo-color, where red indicates high ratio (high potential) and dark blue indicates low ratio (low potential). Yellow through green to light blue represents medium ratio (medium potential).

### Reactive oxygen species (ROS) production assay

The ROS assay was performed using Image-iT LIVE Green Reactive Oxygen Species Detection Kit (I36007; Invitrogen, Grand Island, NY), according to the manufacturers’ protocol. The cells were cultured in 6-well microwell dishes with coverslips for 48 h and further incubated in medium containing M_4_N (80μM) for another 9 to 10 h. After the cells had been washed with warm HBSS/Ca/Mg buffer (Gibco #14025–092; Invitrogen), the cells were incubated in HBSS/Ca/Mg containing 25 μM 5-(and-6)-carboxy-2′,7′-dichlorodihydrofluorescein diacetate (carboxy-H_2_DCFDA) at 37°C in the dark for 30 min. The cells were then washed very gently with HBSS/Ca/Mg buffer three times. The cells were observed through a B29/Zeiss LSM 510 META laser confocal microscope (Carl Zeiss). The cell images were captured with a 488-nm argon-ion laser, because the oxidation product of carboxy-H_2_DCFDA has excitation/emission maxima of approximately 495/529 nm.

### Western blotting

After the cells had been grown in 25-mm^2^ flasks and treated with reagents, they were washed with PBS(-) three times and suspended in RIPA buffer (150 mM NaCl, 50 mM Tris-HCl [pH 8.0], 0.1% SDS, 1% NP40, and 0.5% deoxycholate) supplemented with protease inhibitor cocktail (Calbiochem, San Diego, CA). The sample volumes were adjusted by the total protein amount. Protein assay was performed by Bio-Rad Protein Assay (Bio-Rad Laboratories Inc., Hercules, CA). The samples were resolved by standard SDS-polyacrylamide gel electrophoresis and transferred to nitrocellulose membrane (Amersham Biosciences, Bjorkgatan, Sweden). The membranes were blocked with skim milk and incubated with primary antibodies at 4°C overnight and then with secondary antibody conjugated with horse radish peroxidase at room temperature for 2 h. The signals were detected by western blot chemiluminescence reagent plus (New England Nuclear Life Science Products, Boston, MA). For the assessment of autophagy, the amount of LC3B-II, an indicator of autophagy, was measured by western blotting. Because LC3B-II is synthesized and immediately disintegrated during autophagy, the degradation of this protein needs to be suppressed to measure the net autophagy activity using LC3B-II expression as an indicator [22]. Therefore Bafilomycin A_1_ (100 nM), an inhibitor for degradation of autophagosomes, was added to the experimental system to prevent degradation of LC3B-II.

### Enzymatic assay for caspase-7

The enzymatic assay specific for caspase-7 was performed using a CaspSELECT caspase-7 immunoassay kit (BioVision, Mountain View, CA). This assay is based on the colorimetric assay with DEVD-afc. Because DEVD-afc can be a substrate for either caspase-3 or 7, only caspase-7 is specifically selected from cell extracts by anti-caspase-7 antibody, which is coated on microtiter plates. First the microtiter plates were coated with anti-caspase-7 antibody overnight at 4°C and blocked with a blocking solution for 30 min at room temperature. The cell extracts were applied to the microtiter plates. After washing the plates, the substrate DEVD-afc was added to the plates and incubated for about 3 h. The fluorescence was measured by a microtiter plate fluorescence reader (Infinite 200, Tecan, Mannedorf, Switzerland).

### Northern blotting

RNA was extracted from the cells by Trizol reagent (Invitrogen, Carlsbad, CA). Twenty-five micrograms of RNA per lane was dissolved on the 1.5% agarose gel containing 20 mM NaPO_4_ (pH 6.8) and 6% formaldehyde. RNA was transferred to a Nytran SPC membrane (Sigma-Aldrich, St. Louis, MO). The probe for *BNIP3* was derived from a 199-bp fragment DNA generated by RT-PCR using a SuperScript III First-Strand Synthesis System for RT-PCR (Invitrogen), with the 5'-primer gctcctgggtagaactgcac and 3'-primer gtttcagaagccctgttggt. The PCR fragment was cloned into a topo vector (Invitrogen). After midi-prep, the DNA fragment was excised from the vector and resolved on a 2% agarose gel and purified. The extracted DNA fragment was labeled by ^32^P-αdATP, a Klenow fragment, and random hexagonal primers. After unincorporated ^32^P-αdATP was removed by a Sephadex G-50 spun column, the labeled DNA fragment was used as a probe. After hybridization, the membrane was washed and autoradiographed to a BioMax MR film (Kodak, Rochester, NY).

## Supporting Information

S1 Fig Synergistic cell death by M_4_N combination treatments in various cancer cells. **A:** Synergistic cell death induction in MCF-7 cells. C: control, E: etoposide (30 μM), Ra: rapamycin (30 μM), U: UCN-01 (10 μM), M: M_4_N (80 μM). The cell death was measured by TUNEL assay at 24 h after treatment. Data are presented as mean±SD in triplicate. **B:** A Chou-Talalay plot for TUNEL-positive cell death induced by combination treatments in MCF-7 cells. Combination index (CI) <1, +1, and >1 indicate synergism, additive effect, and antagonism. **C:** Effect of combination treatments of M_4_N with etoposide in OC24 cells. The cell death was measured by TUNEL assay at 24 h after treatment. Data are presented as mean±SD in triplicate. **D:** Effect of combination treatments of M_4_N with rapamycin in various cancer cell lines. The cell death was measured by TUNEL assay. The conditions of each experiment are described in each figure. Data are presented as mean±SD in triplicate. **E:** Effect of combination treatments of M_4_N with UCN-01 in various cancer cell lines. Cell death was examined by TUNEL assay in various cancer cell lines treated with M_4_N and UCN-01 for either 24 or 48 h. The concentration of M_4_N was either 40 or 80 μM. The concentration of UCN-01 was 1, 2, 3, 4, 5, or 10 μM. The exact conditions are shown within each figure. C: control, M: M_4_N, UCN: UCN-01, M+U: M_4_N+UCN-01, U(X): UCN-01 (X μM), MU(Y): M_4_N (80 μM)+UCN-01 (Y μM). Data are presented as mean±SD in triplicate. The data shown here are representatives from multiple independent experiments (For MCF-7, HepG2, LN229, HT29, and OC24 cells, more than three times the experiments were done. For others, two to three times the experiments were done).
(DOCX)

S2 Fig Dose Reduction Index (DRI) for M_4_N combination treatment. **A:** DRI vs Fa plot for M_4_N+etoposide combination treatment in LNCaP cells. **B:** DRI vs Fa plot for M_4_N+rapamycin combination treatment in LNCaP cells. **C:** DRI vs Fa plot for M_4_N+UCN-01 combination treatment in LNCaP cells.
(TIF)

S1 Table The metabolite analysis for LNCaP, AsPC-1, and L428 cells. These cells were treated with M_4_N for 8 h and metabolite contents of the samples were measured by LC/GC mass spectroscopy by Metabolon. Heat map of statistically significant metabolites were profiled and shown in this table. For paired comparisons between the control and M_4_N-treated samples, strongly shaded cells indicate statistically significant differences at p≤0.05 while lightly shaded cells indicate them at 0.05<p<0.10. Inside the table, red indicates that the mean values are significantly higher for that comparison while green values significantly lower. All data normalized using Bradford protein concentration.
(DOCX)
