# Supplementary figures and images for: Tetra-O-Methyl Nordihydroguaiaretic Acid Broadly Suppresses Cancer Metabolism and Synergistically Induces Strong Anticancer Activity in Combination with Etoposide, Rapamycin and UCN-01
Source: PLoS One. 2016 Feb 17;11(2):e0148685. doi: 10.1371/journal.pone.0148685 (PMC4757551; doi:10.1371/journal.pone.0148685)

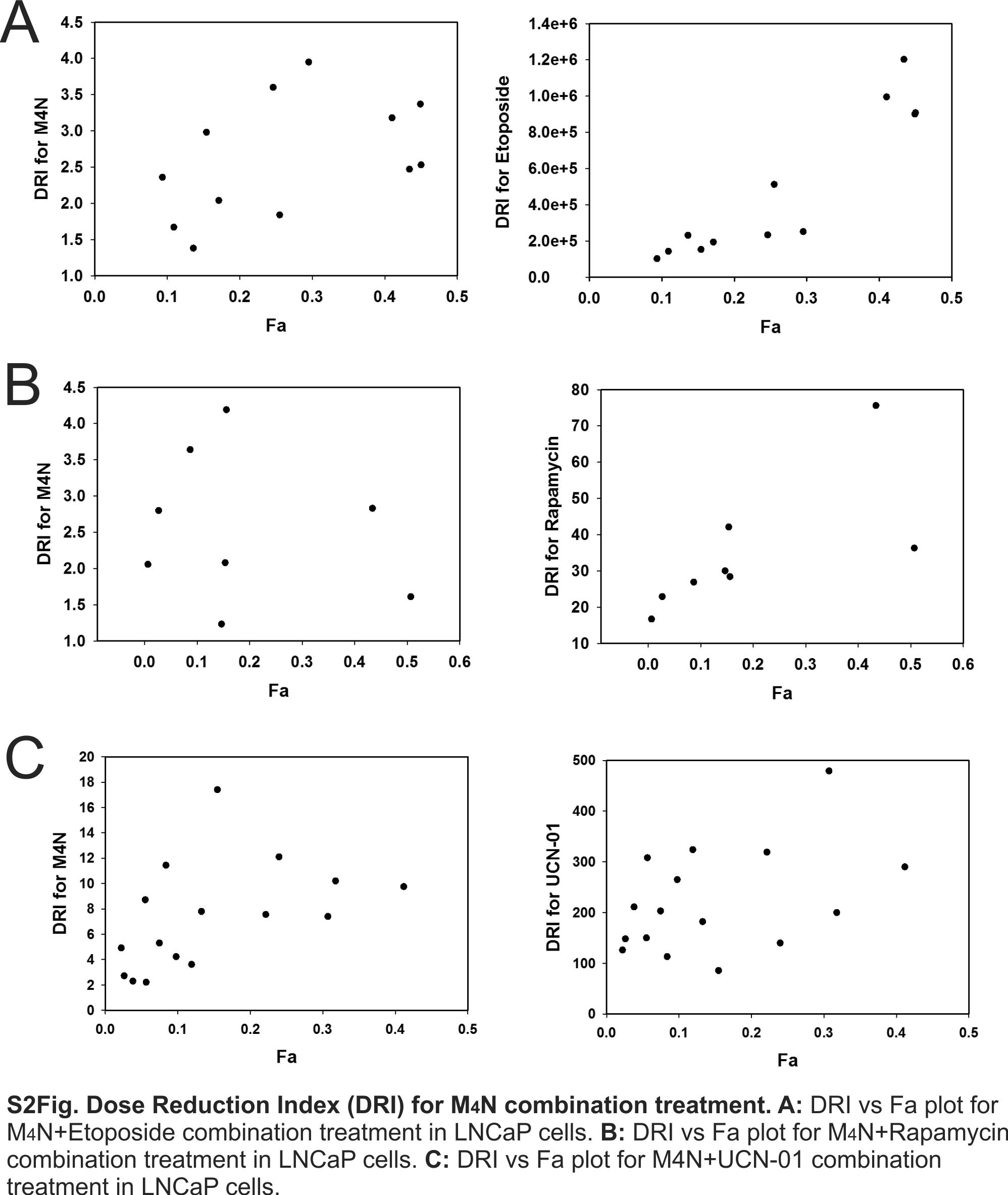

Supplement: S2 Fig — A: DRI vs Fa plot for M4N+etoposide combination treatment in LNCaP cells. B: DRI vs Fa plot for M4N+rapamycin combination treatment in LNCaP cells. C: DRI vs Fa plot for M4N+UCN-01 combination treatment in LNCaP cells. (TIF) [file pone.0148685.s002.tif]
