# Supplementary material for: Tetra-O-Methyl Nordihydroguaiaretic Acid Broadly Suppresses Cancer Metabolism and Synergistically Induces Strong Anticancer Activity in Combination with Etoposide, Rapamycin and UCN-01
Source: PLoS One. 2016 Feb 17;11(2):e0148685. doi: 10.1371/journal.pone.0148685 (PMC4757551; doi:10.1371/journal.pone.0148685)
Supplement: Supplementary file 2 [file pone.0148685.s001.docx]

S1Fig. Synergistic cell death by M_4N combination treatments in various cancer cells.

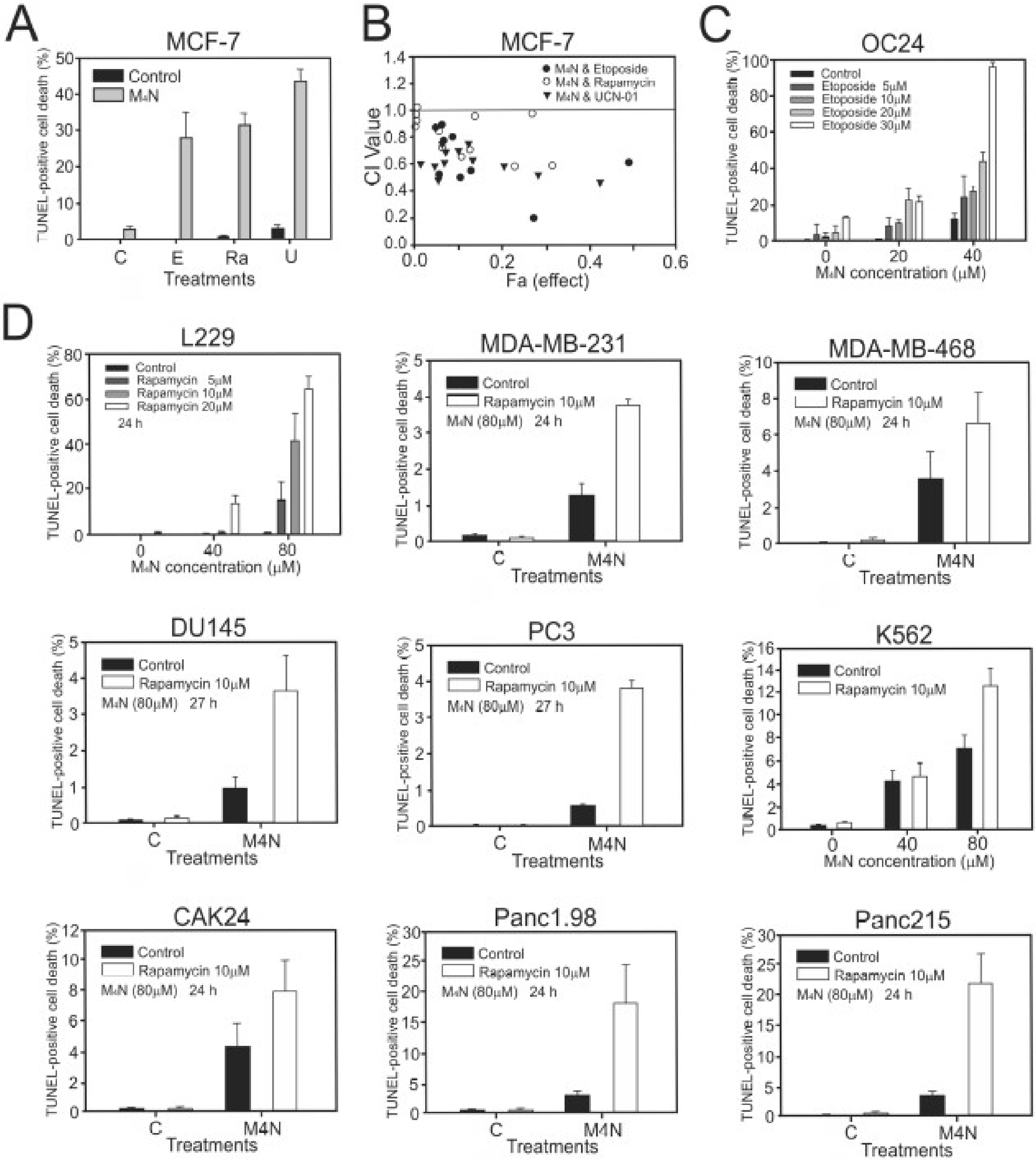

E

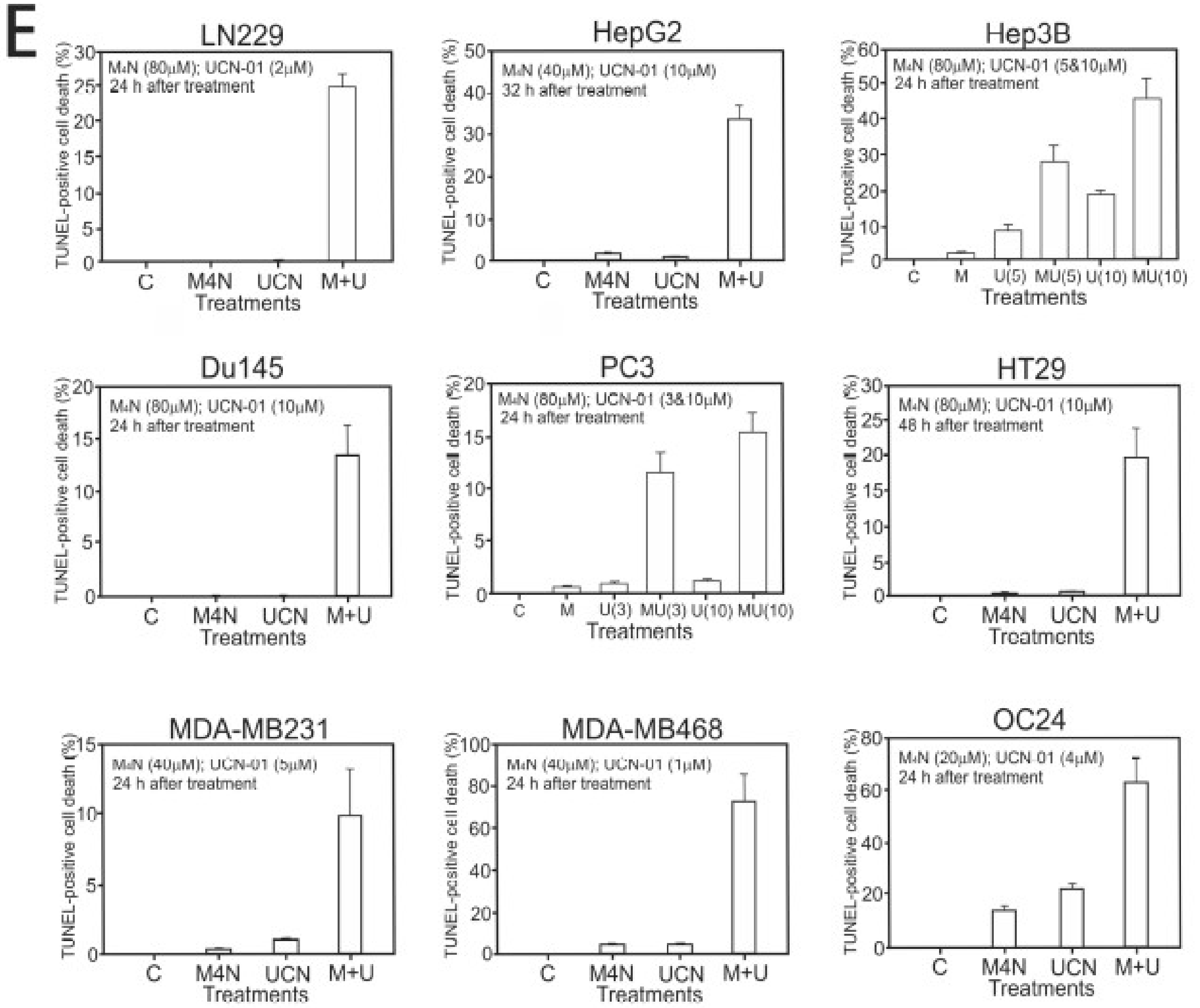

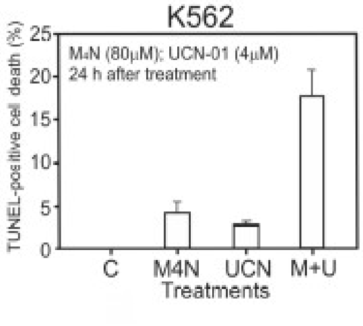

A: Synergistic cell death induction in MCF-7 cells. C: control, E: etoposide (30 μM), Ra: rapamycin (30 μM), U: UCN-01 (10 μM), M: M_4N (80 μM). The cell death was measured by TUNEL assay at 24 h after treatment. Data are presented as mean±SD in triplicate. **B:** A Chou-Talalay plot for TUNEL-positive cell death induced by combination treatments in MCF-7 cells. Combination index (CI) <1, +1, and >1 indicate synergism, additive effect, and antagonism. **C:** Effect of combination treatments of M_4N with etoposide in OC24 cells. The cell death was measured by TUNEL assay at 24 h after treatment. Data are presented as mean±SD in triplicate. **D:** Effect of combination treatments of M_4N with rapamycin in various cancer cell lines. The cell death was measured by TUNEL assay. The conditions of each experiment are described in each figure. Data are presented as mean±SD in triplicate. **E:** Effect of combination treatments of M_4N with UCN-01 in various cancer cell lines. Cell death was examined by TUNEL assay in various cancer cell lines treated with M_4N and UCN-01 for either 24 or 48 h. The concentration of M_4N was either 40 or 80 μM. The concentration of UCN-01 was 1, 2, 3, 4, 5, or 10 μM. The exact conditions are shown within each figure. C: control, M: M_4N, UCN: UCN-01, M+U: M_4N+UCN-01, U(X): UCN-01 (X μM), MU(Y): M_4N (80 μM)+UCN-01 (Y μM). Data are presented as mean±SD in triplicate. The data shown here are representatives from multiple independent experiments (MCF-7, HepG2, LN229, HT29, and OC24 cells, more than three times the experiments were done. For others, two to three times the experiments were done).
