# Supplementary material for: Tetra-O-Methyl Nordihydroguaiaretic Acid Broadly Suppresses Cancer Metabolism and Synergistically Induces Strong Anticancer Activity in Combination with Etoposide, Rapamycin and UCN-01
Source: PLoS One. 2016 Feb 17;11(2):e0148685. doi: 10.1371/journal.pone.0148685 (PMC4757551; doi:10.1371/journal.pone.0148685)
Supplement: Supplementary file 3 [file pone.0148685.s003.docx]

S1Table. The metabolite analysis for LNCaP, AsPC-1, and L428 cells.

SUPER PATHWAY	SUB PATHWAY	BIOCHEMICAL NAME	PLATFORM	M/C		
				LNCaP	AsPC-1	L428
Amino acid-related Metabolites	Amino Acids	glycine	GC/MS	1.31	0.60	1.24
		serine	GC/MS	0.76	1.37	2.25
		threonine	LC/MS pos	1.92	1.59	2.49
		alanine	GC/MS	0.85	1.34	1.39
		aspartate	GC/MS	0.19	0.43	0.45
		asparagine	GC/MS	0.61	1.64	2.14
		glutamate	LC/MS pos	0.74	1.61	1.11
		glutamine	LC/MS pos	2.86	3.18	19.56
		histidine	LC/MS neg	1.33	1.38	2.85
		lysine	GC/MS	1.38	1.21	1.28
		phenylalanine	LC/MS pos	1.03	1.35	2.32
		tyrosine	LC/MS pos	1.36	1.37	2.46
		tryptophan	LC/MS pos	1.33	1.60	2.70
		leucine	LC/MS pos	1.25	1.51	2.96
		isoleucine	LC/MS pos	1.88	1.54	3.02
		valine	LC/MS pos	1.05	1.32	2.53
		methionine	LC/MS pos	1.36	1.58	3.07
		cysteine	GC/MS	0.99	N.A.	N.A.
		cystine	GC/MS	2.16	2.71	2.08
		arginine	GC/MS	1.55	1.14	31.88
		proline	LC/MS pos	1.15	2.08	1.89
	Glutathione Metabolism	5-oxoproline	LC/MS neg	1.89	2.36	4.08
		glutathione, reduced (GSH)	LC/MS pos	0.14	0.76	0.53
		glutathione, oxidized (GSSG)	LC/MS pos	0.17	0.71	0.60
		S-methylglutathione	LC/MS pos	N.A.	1.22	0.81
		S-lactoylglutathione	LC/MS pos	N.A.	0.21	1.00
		S-nitrosoglutathione (GSNO)	LC/MS pos	N.A.	0.36	0.37
Energy	Carbohydrates	glucose	GC/MS	2.73	0.68	1.24
		glucose-6-phosphate (G6P)	GC/MS	0.56	0.72	0.43
		glucose 1-phosphate	GC/MS	N.A.	0.45	0.51
		dihydroxyacetone phosphate (DHAP)	LC/MS neg	N.A.	0.81	1.29
		3-phosphoglycerate	GC/MS	7.08	0.99	1.11
		phosphoenolpyruvate (PEP)	LC/MS neg	N.A.	1.01	1.24
		pyruvate	GC/MS	0.69	3.61	4.58
		lactate	GC/MS	0.11	0.66	0.91
		acetyl CoA	LC/MS neg	0.72	1.00	2.01
		glycerate	GC/MS	2.21	1.09	3.03
	TCA Cycle	citrate	GC/MS	0.74	0.73	0.28
		alpha-ketoglutarate	LC/MS polar	N.A.	1.65	1.18
		succinylcarnitine	LC/MS pos	0.67	0.34	1.00
		succinate	LC/MS neg	0.85	0.15	0.51
		fumarate	GC/MS	0.23	0.70	0.91
		malate	GC/MS	0.27	0.75	0.99
		2-methylcitrate	LC/MS polar	N.A.	1.00	0.82
Lipid-related Metabolites	Carnitine Metabolism	carnitine	LC/MS pos	1.48	1.46	1.96
		deoxycarnitine	LC/MS pos	0.92	0.94	1.34
		acetylcarnitine	LC/MS pos	2.07	3.72	1.09
		hydroxybutyrylcarnitine*	LC/MS pos	N.A.	8.55	5.25
		hexanoylcarnitine	LC/MS pos	N.A.	2.29	0.07
		myristoylcarnitine (14:1)	LC/MS pos	0.42	N.A.	N.A.
		palmitoylcarnitine (16:1)	LC/MS pos	0.44	0.27	0.17
		oleoylcarnitine (18:1)	LC/MS pos	0.04	1.00	0.23
		butyrylcarnitine	LC/MS pos	0.74	14.02	0.47
	Fatty Acid and BCAA metabolism	propionylcarnitine	LC/MS pos	0.08	0.67	0.12
		methylmalonate (MMA)	LC/MS polar	N.A.	0.56	0.21

SUPER PATHWAY	SUB PATHWAY	BIOCHEMICAL NAME	PLATFORM	M/C		
				LNCaP	AsPC-1	L428
Nucleotide	Purine Metabolism, (Hypo)xanthine/inosine containing	xanthine	GC/MS	1.60	1.26	1.59
		xanthosine 5'-monophosphate (xmp)	LC/MS neg	N.A.	1.03	3.44
		xanthosine	LC/MS pos	2.40	1.87	3.16
		hypoxanthine	LC/MS neg	0.75	1.15	1.42
		inosine 5'-monophosphate (IMP)	LC/MS polar	N.A.	1.01	0.74
		inosine	LC/MS neg	0.57	1.13	1.72
	Purine Metabolism, Adenine containing	adenosine 5'-diphosphate (ADP)	LC/MS neg	N.A.	0.35	0.88
		adenosine 5'-monophosphate (AMP)	LC/MS pos	1.02	1.11	1.31
		adenylosuccinate	LC/MS neg	0.27	1.22	1.06
		adenosine	LC/MS pos	0.86	0.69	2.61
		adenine	GC/MS	2.21	0.68	1.96
	Purine Metabolism, Guanine containing	guanosine 5'-triphosphate	LC/MS neg	N.A.	0.56	0.70
		guanosine 5'- monophosphate (GMP)	LC/MS pos	3.63	1.60	1.68
		guanosine	LC/MS pos	1.00	1.52	1.48
		guanine	GC/MS	0.99	1.28	0.97
	Pyrimidine Metabolism, Cytidine containing	cytidine triphosphate	LC/MS neg	N.A.	0.80	0.64
		cytidine diphosphate	LC/MS neg	N.A.	0.64	0.86
		cytidine 5'-monophosphate (5'-CMP)	LC/MS pos	2.46	1.26	1.36
		cytidine	LC/MS pos	2.18	1.53	3.30
	Pyrimidine Metabolism, Uracil containing	uridine 5'-triphosphate (UTP)	LC/MS neg	0.23	0.44	0.48
		uridine 5'-diphosphate (UDP)	LC/MS neg	N.A.	0.37	0.66
		uridine 5'-monophosphate (UMP)	LC/MS neg	1.27	0.93	1.10
		uridine	LC/MS neg	1.08	1.13	1.66
		uracil	GC/MS	1.94	1.22	1.84
	UDP-Sugar	UDP-glucose	LC/MS polar	N.A.	0.54	0.85
		UDP-galactose	LC/MS polar	N.A.	0.66	1.42
		UDP-glucuronate	LC/MS neg	0.80	0.74	0.47
		UDP-acetylglucosamine, UDP-acetylgalactosamine	LC/MS neg	0.67	0.44	0.49
		UDP-N-acetylgalactosamine	LC/MS polar	N.A.	0.54	0.49

These cells were treated with M_4N for 8 h and metabolite contents of the samples were measured by LC/GC mass spectroscopy by Metabolon. Heat map of statistically significant metabolites were profiled and shown in this table. For paired comparisons between the control and M_4N-treated samples, strongly shaded cells indicate statistically significant differences at $p=0.05$ while lightly shaded cells indicate them at $0.05<p<0.10$. Inside the table, red indicates that the mean values are significantly higher for that comparison while green values significantly lower. All data normalized using Bradford protein concentration.
